# Clinical outcomes in patients relapsed/refractory after ≥2 prior lines of therapy for follicular lymphoma: a systematic literature review and meta-analysis

**DOI:** 10.1186/s12885-023-10546-6

**Published:** 2023-01-23

**Authors:** Steve Kanters, Graeme Ball, Brad Kahl, Adriana Wiesinger, Eve H. Limbrick-Oldfield, Akshay Sudhindra, Julia Thornton Snider, Anik R. Patel

**Affiliations:** 1 RainCity Analytics, Vancouver, BC Canada; 2 Gilead Sciences Canada, Inc., Mississauga, Canada; 3 Oncology Division, Department of Medicine, Washington University School of Medicine in St. Louis, St. Louis, MO USA; 4 Kite, A Gilead Company, London, UK; 5 Kite, A Gilead Company, Santa Monica, USA

**Keywords:** Relapsed/refractory follicular lymphoma, Clinical outcomes, Systematic literature review, Meta-analysis

## Abstract

**Background:**

Patients with follicular lymphoma (FL) can have high response rates to early lines of treatment. However, among FL patients relapsed/refractory (r/r) after ≥2 prior lines of therapy (LOT), remission tends to be shorter and there is limited treatment guidance. This study sought to evaluate the clinical outcomes for r/r FL after ≥2 prior LOT identified through systematic literature review.

**Methods:**

Eligible studies included comparative or non-comparative interventional or observational studies of systemic therapies among adults with FL r/r after ≥2 prior LOT published prior to 31st May 2021. Prior LOT must have included an anti-CD20 monoclonal antibody and an alkylating agent, in combination or separately. Overall response rate (ORR) and complete response (CR) were estimated using inverse-variance weighting with Freeman-Tukey double-arcsine transformations. Kaplan-Meier (KM) curves for progression-free survival (PFS) and overall survival (OS) estimated by reconstructing digitized curves using the Guyot algorithm, and survival analyses were conducted, stratified by ≥2 prior LOT and ≥ 3 prior LOT groups (as defined in the source material). Restricting the analyses to the observational cohorts was investigated as a sensitivity analysis.

**Results:**

The analysis-set included 20 studies published between 2014 and 2021. Studies were primarily US and/or European based, with the few exceptions using treatments approved in US/Europe. The estimated ORR was 58.47% (95% confidence interval [CI]: 51.13–65.62) and proportion of patients with CR was 19.63% (95% CI: 15.02–24.68). The median OS among those ≥2 prior LOT was 56.57 months (95% CI: 47.8–68.78) and median PFS was 9.78 months (95% CI: 9.01–10.63). The 24-month OS decreased from 66.50% in the ≥2 prior LOT group to 59.51% in the ≥3 prior LOT group, with a similar trend in PFS at 24-month (28.42% vs 24.13%).

**Conclusions:**

This study found that few r/r FL patients with ≥2 prior LOT achieve CR, and despite some benefit, approximately 1/3 of treated patients die within 24 months. The shorter median PFS with increasing prior LOT suggest treatment durability is suboptimal in later LOT. These findings indicate that patients are underserved by treatments currently available in the US and Europe.

**Supplementary Information:**

The online version contains supplementary material available at 10.1186/s12885-023-10546-6.

## Background

Non-Hodgkin lymphoma (NHL) is the eleventh most commonly diagnosed malignancy in the world and accounts for the eleventh highest cancer-related mortality [1]. It is estimated that in 2020 there were 544,352 cases of NHL diagnosed globally, and more than 259,793 deaths among patients afflicted by this malignancy [1]. The primary risk factor for NHL is older age, with greater than half of patients being diagnosed at age 65 or older [2]. In the coming decades the generational aging in many areas of the world is likely to lead to a subsequent increase in global NHL cases.

NHL can be broadly categorized into aggressive and indolent NHL (iNHL) based on rate of progression [3]. iNHL is typically a slow growing cancer that is often asymptomatic and discovered incidentally. Approximately one-third of malignant lymphomas are iNHL [4], which are further subdivided by histology, with follicular lymphoma (FL) and marginal zone lymphoma (MZL) being the most commonly diagnosed histologies. Notably, despite its relatively high incidence and prevalence, FL is generally considered to be incurable with standard front-line therapies [5].

The introduction of front-line chemoimmunotherapy, employing an alkylator and anti-CD20 monoclonal antibody combination, such as R-CHOP (rituximab, cyclophosphamide, doxorubicin hydrochloride, vincristine and prednisone), has led to a nearly 100% overall response rate among first-line FL patients [6]. Approximately 20% of FL patients are expected to experience disease relapse within 2 years of treatment [2], and the disease tends to become increasingly refractory to treatment with successive each line of therapy [7]. Among relapsing patients, remission tends to also become shorter with additional line of therapy [8].

There are numerous treatments that have recently come to market or are currently under study. Anti-CD19 chimeric antigen receptor T cells (CAR-T) have shown promise in patients with B-cell cancers [9]. Following the approval of CAR-T for adult patients with r/r diffuse large B-cell lymphoma (DLBCL) and primary mediastinal large B-cell lymphoma (PMBCL) after 2 or more lines of systemic therapy, CAR-T was recently approved for r/r FL [10]. Clinical trials are ongoing for several CAR-T therapies in r/r FL [11, 12]. Other novel therapies that have been investigated in r/r FL [13, 14] include the anti-CD20/CD3 bispecific antibody odronextamab, and the PI3K inhibitor idelalisib. Many of the recent and ongoing trials are non-comparative in nature, so better understanding the treatment landscape for r/r FL patients would help to contextualize their results.

Critically, despite the advent of newer therapies being added to the r/r iNHL armamentarium, there is a need for data on the impact of currently available agents on long-term prognosis for patients with r/r iNHL. The current study therefore utilized a comprehensive methodological approach to evaluate and summarize the clinical outcomes of currently available agents through a systematic literature review (SLR) and meta-analysis of treatments available for therapy in Europe and the US for r/r FL patients having been failed by ≥2 prior lines of therapy.

## Methods

### Systematic literature review

A comprehensive systematic search of the literature was conducted on 31 March 2021 using the following databases on the Ovid platform: Medical Literature Analysis and Retrieval System Online (MEDLINE), Excerpta Medica database (EMBASE), and Cochrane Central Register of Controlled Trials (Additional file: Tables S1, S2, S3). Searches were conducted in accordance with recommendations from the Cochrane Collaboration, National Institute for Health and Care Excellence (NICE) guidance, Institut für Qualität und Wirtschaftlichkeit im Gesundheitswesen (IQWiG in Germany). Manual searches were also undertaken of relevant conference proceedings over the previous 2 years, as well as international clinical trial databases, to identify additional eligible studies.

Eligible studies for the SLR were among adults (aged ≥18 years) with r/r iNHL after failure of two or more lines of therapy. For the purpose of this study, the analysis-set was further reduced to r/r FL patients as discussed in further detail below. Randomized control trials, non-randomized trials, observational studies and registries were all eligible study designs. Eligible interventions were any approved for treatment in the US or Europe, best supportive care or placebo. Here too, the SLR scope was broad, including genetic therapies and therapies approved for other iNHL indications (e.g., ibrutinib is approved for marginal zone lymphoma and other iNHL, but not for FL). The full study eligibility criteria, defined in terms of the population, interventions, comparisons, outcomes, and study design (PICOS), are outlined in Additional file 1: Table S4.

**Fig. 1:**
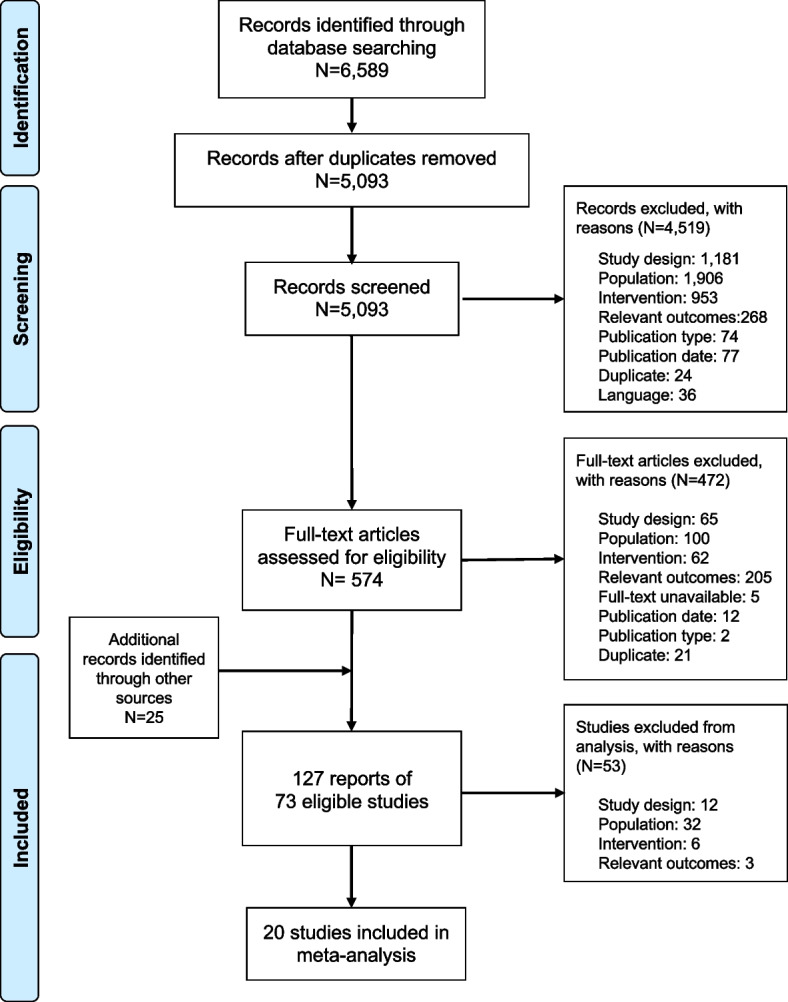
PRISMA flow diagram

Two reviewers, working independently, reviewed all abstracts and proceedings identified in the searches according to the selection criteria, with the exception of outcome criteria which were adjudicated during full-text screening. Eligible studies then underwent full-text screening by the same two reviewers, and full-text studies that met the inclusion criteria were identified for data extraction. Any disagreement between the two reviewers was adjudicated and resolved by a third reviewer. This process is detailed in the PRISMA [15] flow diagram (Fig. 1).

Data on study characteristics, interventions, patient characteristics, and outcomes for the final list of included studies was extracted by the two independent reviewers. Since direct access to study data was not available for time-to-event outcomes, survival curves were digitally extracted using the DigitizeIt software. These were then used to generate pseudo-individual patient-level data by applying the Guyot algorithm with numbers at risk tables [16]. Time-to-event data from the reconstructed survival curves were extracted by one reviewer and then independently verified by the second reviewer.

Given the mixed study designs eligible for the evidence base (i.e., the eligibility of both randomized and non-randomized studies), the quality assessment for the evidence base was performed using the Downs and Black checklist [17]. This study quality tool is well established and lends itself to all eligible study designs, which allowed for a single assessment tool to be used for all studies.

Study selection for inclusion in the analysis set was conducted in two steps. First, a feasibility-assessment-set was identified by reducing patients to the scope of the project at hand. Studies including small lymphocytic lymphoma, lymphoplasmacytic lymphoma, MZL only or transformed FL/MZL were removed, unless subgroups excluding these patients were available. Studies restricted to Grade 1 and 2 FL were also excluded from analyses. One study explicitly included Grade 3b patients [18], which, after further review, a judgment was made that the few Grade 3b patients included in the trial would have negligible impact on the outcomes of interest, and thus this study was included in the analysis set. Studies examining CAR-T therapy were also removed as CAR-T did not represent an available treatment modality at the time of analysis. Second, studies were further restricted following the results of the feasibility assessment. The analysis set was restricted to sample sizes of at least 20 patients because a few studies reporting on FL as a subgroup had very small sample sizes (often below 5) that led to high levels of heterogeneity.

### Statistical analyses

A frequentist meta-analysis approach was used for the ORR, CR, PFS and OS outcomes and a Bayesian approach was used in meta-analysis of the digitized Kaplan-Meier curve data for the time-to-event outcomes. Treatments identified from studies that met the inclusion criteria were simplified for the purpose of analysis into the following categories: standard of care (SoC), PI3k-δ inhibitors, Lenalidomide + Rituximab, Bortezomib + Rituximab, Obinutuzumab + Benda, 90Y + Anti-CD20 combination, Autologous stem cell transplant (SCT), and Allogeneic SCT. The evidence base included data from three studies [7, 19, 20] that included a heterogenous sampling of both treatments and patient populations. These were considered to be representative of typical care and thus were dubbed to be *representative cohorts*. The most common treatments were anti-CD20 monoclonal antibodies, with or without chemotherapy [21, 22], and PI3k-δ inhibitors [23–26].

All meta-analyses using single summary statistics of proportions were based on dichotomous outcomes: ORR and CR. For the analysis of each of these outcomes, inverse-variance meta-analyses were used. The Freeman-Tukey double arcsin transform was used throughout to ensure stability in the extreme proportion values (near 1 or 0). Our review of the data revealed multiple instances of observed proportions of 1, so this was deemed necessary. The analyses were stratified by the treatment categories outlined above. Both fixed- and random-effects were used within the strata, but random-effects were not used between them. The results from each stratum were combined using a weighted mean with relative sample size as the weight. Weights were designed to sum up to 1 to ensure an unbiased estimate. Heterogeneity within strata was assessed using the I^2^ statistic.

Meta-analyses for the digitized Kaplan-Meier survival curves, for both OS and PFS, were analyzed in both the frequentist and Bayesian framework. Bayesian analyses used non-informative prior distributions and were based on methods for network meta-analyses of survival data using a multidimensional treatment effect as an alternative to the synthesis of the constant hazard ratios, as developed be Ouwens et al. [27] and Jansen [28]. Namely, the hazard functions of the interventions in a trial were modeled using known parametric survival functions or fractional polynomials. Given the non-comparative nature of this evidence base, a simple version of the model introduced by Jansen was used for the meta-analyses of OS and PFS [28, 29].

Of note, patients included in the representative cohorts were followed from one line to the next and as a result, observations were not fully independent for OS and PFS. In addition, restricting analyses to include only patients in their third line of treatment was deemed more detrimental than having repeated measures among some patients, and thus no such restrictions were implemented. Where permitted by the evidence, analyses also included those patients receiving a fourth line or more of treatment.

For Bayesian analyses, the deviance information criterion (DIC) was used to compare the goodness-of-fit of competing survival models [30]. A difference in DIC of approximately 5 points was considered meaningful and, in the case of survival models, the hazard functions were visually inspected for over-fitting [16]. The parameters of the different models were estimated using a Markov Chain Monte Carlo method implemented in the JAGS software package. A first series of 20,000 iterations from the JAGS sampler were discarded as ‘burn-in’, and the inferences were based on an additional 40,000 iterations using two chains. For all analyses, model convergence was assessed through trace plots, density plots and Gelman-Rubin-Brooks (shrink factor) plots [31].

The patient population in the primary analyses were restricted to patients with FL receiving therapies other than transplant because: a) this treatment modality represents a very different intervention to those being studied; b) the SCT study populations tended to be significantly younger and healthier; and c) these studies appeared to be overrepresented in the evidence base. Furthermore, as these studies only reported on patients who survived through to SCT, these studies were at risk of immortal time bias. The primary model also excluded off-label treatments for FL, as these were considered atypical. A second model included only study cohorts that were representative of care. Two supplemental models included a) off-label treatments, and b) only SCT studies. The viability of each model depended upon data availability (Additional File 1: Table S5).

## Results

From the 6589 citations identified in the database and 25 through conference proceedings searches, a total of 126 publications describing 72 unique studies were eligible for inclusion in the SLR iNHL evidence base. The analysis-set excluded studies for the following reasons: 32 on the basis of population [MZL (3 studies), CLL/SLL/LPL/Transformed FL or Grade 3b FL (9 studies), FL grade 1 and 2 only (2 studies) and older studies guaranteed to have no prior anti-CD20 (18 studies)], 3 for outcomes, 6 for intervention and 12 for study design, including small sample sizes. The complete flow diagram leading to the selection of the SLR evidence base is presented in Fig. 1.

Of the 20 studies included in the analysis-set, 9 were single-arm clinical trials [21, 24, 32–38], 9 were retrospective cohort studies [7, 19, 22, 23, 25, 39–42] and 2 were prospective cohort studies [20, 43]. Two of the single arm trials were Phase I dose escalation studies, whilst the rest were Phase II non-comparative trials. Studies were conducted in a variety of countries, with nearly half being conducted in the US and the majority conducted in the US and/or Europe. Further study characteristics, including location, are presented in Table 1. We assessed the risk of patient overlap between the cohort studies, and concluded some overlap was possible, but due to the different geographies, treatment regimens, treatment centers and dates of patient inclusion, this overlap was minimal and not of concern. The quality assessment of the included studies, performed using the Downs and Black checklist [17], rated 13 studies as fair and 7 studies as poor (Additional file 1: Table S6). However, studies of poor quality tended to be non-comparative, for which a considerable number of items on the check-list are non-applicable (i.e., it would be reasonable to qualify these as fair quality). The majority of the studies reported response criteria used (Additional File 1: Table S7), with the 2007 IWG revised guidelines being the most frequently used [44]. However four studies used the 1999 IWG criteria, [45] and UNITY-NHL used Lugano classification [46].

**Table 1:** Study characteristics of included studies

Study	Location	Year	Treatment	Study design	N	Median follow up (months)	Follow-up range
Andorsky 2019 [23]	US	2019	Idelalisib	Retrospective cohort study	54	18.6	0.6–49.5
Assouline 2020 [32]	US, Australia, South Korea, Canada, Europe	2020	Mosunetuzumab	Phase I trial	62	14.4	–
Batlevi 2020 [7]	US	2020	Representative cohort	Retrospective cohort study	299	87.6	2.4–200.4
CHRONOS 1 part B [33]	Europe, Asia, US, Australia, New Zealand	2017	Copanlisib	Phase II trial	104	6.69	0.23–24.01
DAWN [34]	Europe, Asia, US, South America, Australia	2018	Ibrutinib	Phase II trial	110	27.7	1.1–37.1
DELTA [24]	Europe, US	2014	Idelalisib	Phase II trial	72	33.9	1.2–81.4
ELM-1 [21]	Europe, US, Canada, Australia, Asia	2020	Odronextamab	Phase I trial	28	6.8	1.0–22.1
Evens 2013 [43]	US	2013	Allo-SCT	Prospective cohort study	184	48	N/R
EZH [35]	Europe, US, Canada, Australia, Taiwan	2018	Tazemetostat	Phase II trial	99	N/R	N/R
Fuji 2020 [19]	Japan	2020	Representative cohort	Retrospective cohort study	41	89.64	28.56–134.40
Ito 2013 [39]	Japan	2013	Allo-SCT	Retrospective cohort study	30	21.4	0.4–165.4
Khouri 2008 [36]	US	2008	Allo-SCT	Phase II trial	47	107	72–142
Laport 2016 [37]	US	2016	Allo-SCT, Auto SCT	Phase II trial	62	47	30–73
Link 2019 [20]	US	2019	Representative cohort	Prospective cohort study	438	96	0.24–124.8
Lunning 2016 [40]	US	2016	Auto-SCT	Retrospective cohort study	44	60	21.8–96
Muntanola 2020 [22]	Spain	2020	R-ESHAP	Retrospective cohort study	28	N/R	N/R
Robert 2019 [25]	France	2019	Idelalisib	Retrospective cohort study	24	23	20–24
Sesques 2020 [41]	France	2020	Auto-SCT	Retrospective cohort study	61	105.6	27.6–291.6
UNITY-NHL [38]	Europe, US, Australia, Korea	2021	Umbralisib	Phase II trial	117	27.5	20.9–37.1
Vose 2008 [42]	US	2008	Auto-SCT	Retrospective cohort study	108	72	12–192

### Response outcomes

**Fig. 2:**
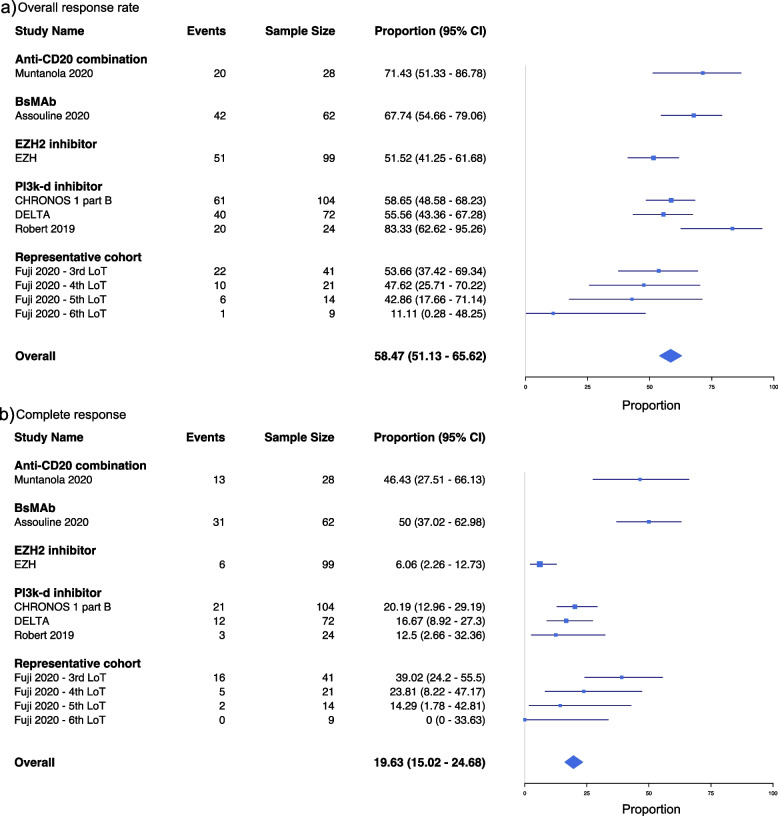
Meta-analysis of response outcomes. BsMAb, bispecific monoclonal antibody; CI, confidence interval; EZH2. Enhancer of zeste homolog 2; PI3k-d Phosphoinositide 3-kinase delta

The meta-analysis revealed an overall ORR of 58.47% (CI: 51.13–65.62%) and an overall CR of 19.63% (CI: 15.02–24.68%) (Fig. 2**)**. As can be observed, there was notable heterogeneity between studies. In the supplementary model (Additional File 1: Table S8, Fig. S1), the inclusion of off-label treatments found similar results as the primary analyses, with an ORR of 52.40% (CI: 46.37–58.39%), CR of 17.46% (CI: 13.59–21.70%). Off-label treatments included ibrutinib, which is only approved for other iNHL indications by both the EMA and FDA, odronextamab, which is not yet approved globally, and umbralisib, which is aimed at MZL but indicated in the US for 4 L+ patients only (EMA has granted a waiver to all mature B cell malignancies).

### Time-to-event outcomes

**Fig. 3:**
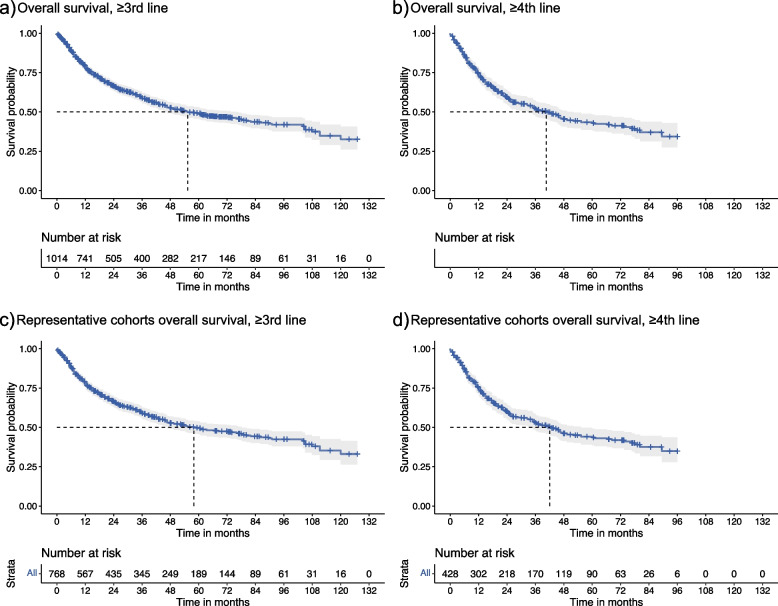
Summary KM curves for overall survival. Dotted line shows median, shaded area = 95% CI

**Fig. 4:**
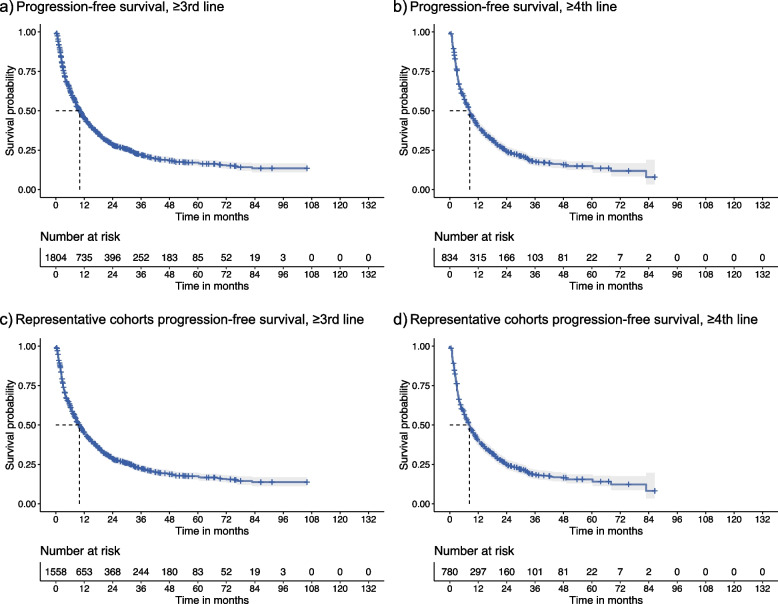
Summary KM curves for progression-free survival. Dotted line shows median, shaded area = 95% CI

The Bayesian and frequentist analyses provided similar results with regard to clinical outcomes, with results for each approach presented in Table 2 and Table 3, respectively. The selected fractional polynomial parameters for each model are shown in Table S9. Summary KM curves are also presented (Figs. 3 and 4) for OS and PFS for those in the 3rd or greater LOT, 4th or greater LOT, and subsequent representative cohorts. With regard to OS, the main analyses and representative cohorts were similar in magnitude. A notable decrease in the median OS was evident among those in the 4th or greater LOT as compared to the 3rd or greater LOT (39.89 months vs. 56.57 months), suggesting that the data from the 3rd or greater LOT group may be attenuated by the inclusion of the latter group. A similar pattern was observed in the representative cohorts being treated in these later LOTs. Supplementary analyses of patients undergoing SCT showed a significantly higher OS, with a median OS of 93.9 months (CI: 81.8–107.96) in the 3rd or greater LOT (Additional File 1: Table S10, Fig. S2, S3).

**Table 2:** Median OS and PFS using pseudo IPD from Kaplan-Meier curves

Population	Frequentist analyses		Bayesian fractional polynomial meta-analyses	
	Median OS Months (95% CI)	Median PFS Months (95% CI)	Median OS Months (95% CI)	Median PFS Months (95% CI)
≥3rd line	55.34 (46.60, 76.00)	10.09 (9.25, 11.10)	56.57 (47.8–68.78)	9.78 (9.01–10.63)
≥4th line	40.63 (32.58, 52.12)	8.41 (7.47, 9.48)	39.89 (31.79–51.94)	8.11 (7.3–9.04)
Representative cohorts ≥3rd line	57.94 (46.60, 78.41)	9.99(8.62, 11.10)	58.67 (48.56–72.47)	9.43 (8.57–10.4)
Representative cohorts ≥4th line	42.02 (34.80, 55.34)	8.33 (7.11, 9.56)	41.63 (32.93–55.12)	7.9 (7.02–8.93)

*Frequentist results and median as estimated using Bayesian fractional polynomials

**Table 3:** OS and PFS at 18 months and 24 months using frequentist meta-analysis

Population	OS at 18 m %(95% CI)	PFS at 18 m % (95% CI)	OS at 24 m % (95% CI)	PFS at 24 m (95% CI)
≥3rd line	71.49 (68.68, 74.42)	35.37 (33.15, 37.75)	66.50 (63.54, 69.60)	28.26 (26.15, 30.55)
≥4th line	65.07 (60.84, 69.59)	31.36 (28.30, 34.75)	59.51 (55.12, 64.24)	24.13 (21.29, 27.35)
Representative cohorts ≥3rd line	70.92 (67.71, 74.29)	35.52 (33.16, 38.05)	66.45 (63.08, 69.99)	28.42 (26.18, 30.85)
Representative cohorts ≥4th line	65.07 (60.84, 69.59)	31.36 (28.30, 34.75)	59.51 (55.12, 64.24)	24.13 (21.29, 27.35)

A similar pattern of results was observed with regard to PFS, with a median PFS of 9.78 (CI: 9.01–10.63) months among those receiving their 3rd or greater LOT as compared 8.11 (CI: 7.3–9.04) months among the 4 or greater LOT group. Observations in the representative cohorts (9.43 months vs. 7.9 months) suggested a similar pattern of attenuation in the 3rd or greater LOT results. In the supplementary analyses, the inclusion of off-label treatments did not have a marked effect on the median PFS (9.86 (CI: 9.16–10.67) months), whereas those patients undergoing SCT were reported to have longer median PFS of 38.58 (CI: 31.37–47.94) months.

The 24-month OS decreased from 66.50% (CI: 63.54–69.60) in the ≥2 prior LOT group to 59.51% (CI: 55.12–64.24) in the ≥3 prior LOT group, with a similar trend in PFS at 24-month (28.26% vs 24.13%). Once again, a similar pattern of results was observed in the representative cohorts, with a reduction in OS from 66.45 to 59.51% and PFS from 28.42 to 24.13%.

## Discussion

The purpose of this study was to determine the therapeutic effects of treatments available in Europe and the US for r/r FL patients having been failed by ≥2 prior lines of therapy. Our SLR identified multiple studies including large representative cohorts. Results of the analyses point to a number of unmet needs in this population. The overall response rate was low (57%), despite the inclusion of more studies of recent-to-market treatments (e.g., PIK3-δ and EZH2 inhibitors) that are less commonly used in real-world settings. The median progression-free survival time was also low (median: 9 months), indicating an unmet need. The median survival time was high (59 months), which reflects the indolent nature of the disease. This study provides important context for the results of clinical trials and future studies in r/r FL.

The search methodology we employed was comprehensive and identified studies that were geographically diverse and featured a mix of retrospective cohort studies and non-randomized single-arm clinical trials; thus, the poor clinical outcomes identified in this study emphasize the significant unmet need among this patient population being treated with existing therapeutic agents. This important insight into the limited efficacy of therapies currently available in the treatment of r/r FL can also be used as a point of comparison for ongoing clinical trials of CAR T-cell therapies in this disease space. For example, Jacobsen and colleagues reported a 95% ORR and an 81% CR among FL patients in the ZUMA-5 trial [47] and Fowler and colleagues reported an 85% ORR and a 69% CR among FL patients in the ELARA trial [48]. Whereas there exist several potential differences in the population examined in this review and those enrolled in the ZUMA-5 and ELARA trials, the response rates reported in these clinical trials were notably higher than those found in the SLR reported here. These population differences may explain the differences in overall survival. Jacobson and colleagues reported a > 80% survival at 24 months compared to the 57% noted in this review; although the median OS in this trial has not been reached and thus conclusions regarding OS must be tempered.

The natural disease course of iNHL, with its relapsing and refractory nature and limited treatment options, particularly in later lines of therapy, can exert significant burden on patients and their families. The uncertainty associated with long-term prognosis, ongoing treatment regimens and their toxicities, and frequent interactions with the medical establishment, can all lead to diminished quality of life and poorer psychosocial outcomes [49, 50]. Given the limited treatment efficacy observed in this study, and the prolonged disease course associated with iNHL, it may be prudent for healthcare providers to engage in shared decision making with patients and select treatment regimens that strike a balance between minimizing tumor burden and toxicity while also maximizing quality of life [51, 52].

The current study possesses both strengths and limitations that should be noted. Among the strengths is the robustness of the survival analysis, where sophisticated methods were used to maximize the inclusion of information available in the literature. Through digitization of survival curves, pseudo individual patient data were obtained which allowed for estimation of the entire survival curve all at once, rather than only at specific time points.

In terms of limitations, firstly the sample population was non-representative. Importantly, the goal of this study was to characterize a patient population and not to estimate a comparative treatment effect, and thus measures were taken to create a sample that is reflective of the general population. Despite these efforts, such a condition was not met by our evidence base, with the most notable difference being that concerning treatments received. Generally, there was an over-representation of modern treatments (e.g., SCTs and PI3K-δ inhibitors) and a subsequent under-representation of anti-CD20 and/or chemotherapies that remain common (e.g., R-CHOP). This non-representativeness was further seen in response outcomes, where PI3K-δ inhibitors were heavily represented as only one representative cohort study reported response outcomes. In these analyses response outcomes may be biased towards these more recently approved treatments. The exclusion of SCT studies was necessary due to the immortal time bias introduced and the lack of intention-to-treat analyses in a setting where many patients do not meet criteria to receive treatment. Nonetheless, it is important to note that the representative cohort analyses did include SCT patients, and thus were included in the main analysis. Also, SCT studies reported high survival rates, suggesting SCT is an effective treatment.

A second important limitation pertains to the representative cohort studies that were included in the analyses. A series of three recent studies [7, 19, 20] were the primary sources of insight here. Notably, the results of these studies were presented by line of treatment such that some patients provided data at multiple points. Given the aggregate nature of the data, it was impossible to disaggregate the data to adjust for the repeated measures among patients. In an ideal situation, patients progressing from 3rd line to 4th line would be censored for time-to-event analyses at the time of switch. While this study could have restricted the analyses to a specific line only, it wouldn’t have allowed for inference on the target population, namely 3 L+ r/r FL patients. The potential bias due to repeated measures was deemed less detrimental than the removal of later lines altogether. The issue of repeated measures was reduced for PFS relative to OS because the events were unlikely to be shared across lines of therapy. Typically progression leads to a subsequent change of line of therapy.

Finally, response assessment differed both within and between studies. For the representative cohort studies, response assessment criteria were not reported. For the studies that did report criteria, the 1999 IWG-NHL criteria [45], the 2007 IWG-NHL criteria [44] and the Lugano classification [46] were all used, dependent upon when patients received the index treatment, and what imaging was available. The imaging modality used for response assessment may lead to differences in CR rates, with CT based assessment resulting in lower CR than PET-CT based assessment. This potential bias should be considered when interpreting the CR results.

In conclusion, this comprehensive systematic literature review and meta-analysis further emphasize the significant unmet need among those patients diagnosed with r/r FL patients being failed by ≥2 prior lines of therapy. The low to moderate rates of CR and ORR, as well as the short median time to progression, highlight the need for novel treatment options to be developed and approved among this patient population.

## Supplementary Information

**Additional file 1: Table S1.** Embase search strategy. **Table S2.** Cochrane Central Register of Controlled Trials search strategy. **Table S3.** MEDLINE search strategy. **Table S4.** Study selection criteria to identify trials for the systematic literature review. **Table S5.** Studies included in each meta-analysis. **Table S6.** Study quality assessment results. **Table S7.** Response criteria used for each study included in the meta-analysis. **Table S8.** Meta-analysis of response outcomes, separated by treatment category, a. Main analysis, b. With inclusion of off-label treatments. **Table S9.** Model selection across the Bayesian analyses. **Table S10.** Time-to-event meta-analysis results for supplemental models. **Fig. S1.** Meta-analysis of response outcomes, including off-label treatments, A) Overall response rate, B) Complete response. **Fig. S2.** Pooled KM curves for supplemental model including off-label treatments, A) PFS 3rd line plus, B) OS 3rd line plus, **Fig. S3.** Pooled KM curves for supplemental model including only SCT studies, A) PFS 3rd line plus, B) OS 3rd line plus.

## Data Availability

The data analyzed in this study were obtained from publicly available sources. The following sources were used: Medical Literature Analysis and Retrieval System Online (MEDLINE), Excerpta Medica database (EMBASE), and Cochrane Central Register of Controlled Trials.
