# Supplementary material for: Clinical outcomes in patients relapsed/refractory after ≥2 prior lines of therapy for follicular lymphoma: a systematic literature review and meta-analysis
Source: BMC Cancer. 2023 Jan 23;23:74. doi: 10.1186/s12885-023-10546-6 (PMC9869623; doi:10.1186/s12885-023-10546-6)

**Additional file 1**

**Supplementary methods: Bayesian survival analyses**

Meta-analyses for the digitized Kaplan-Meier survival curves, for both OS and PFS, were analyzed in the Bayesian framework. Bayesian analyses involve prior distributions, a likelihood statement and a model, whereby a posterior distribution is obtained by updating the prior distribution according to the observed evidence. All analyses used non-informative priors with a normal distribution. Traditional meta-analyses for survival outcomes center around hazard ratios. The hazard function describes the instantaneous event (e.g. death) rate at any point in time. Here the hazard functions of the interventions in a trial are modeled using known parametric survival functions or fractional polynomials. Given the non-comparative nature of this evidence base, a simple version of the model introduced by Jansen will be used for the meta-analyses of OS and PFS (Jansen JP. Network meta-analysis of survival data with fractional polynomials. BMC Med Res Methodol. 2011;11:61. doi:10.1186/1471-2288-11-61).

For these analyses, data were transformed from the pseudo-individual patient data (i.e., each patient is assigned an event status and a time of event) to a series of *q* consecutive intervals over the follow-up period: $\left[ t_{1},t_{2} \right], \left( t_{2},\left. t_{3} \right] \right.,\ldots,\left( t_{q},\left. t_{q+1} \right] \right.$ with $t_{1}=0$. For each time interval *t*=1,2,3,…,*.q,* extracted survival proportions were used to calculate the patients at risk at the beginning of that interval and incident number of deaths. A binomial likelihood distribution of the incident events for every interval can be described according to:

$$r_{im} \sim Bin(p_{im}, n_{im})$$

where $r_{jm}$ is the observed number of events in the m^th^ interval ending at time point $t_{m+1}$ in study *j*. $n_{jm}$ is the number of subjects at risk just before the start of that interval, adjusted for the subjects censored in the interval. $p_{jm}$ is the corresponding underlying event probability. When the time intervals are relatively short, the hazard rate $h_{jt}$ at time point *t* for treatment *k* in study *j* can be assumed to be constant for any time point within the corresponding m^th^ time interval. The hazard rate corresponding to $p_{jt}$ for the m^th^ interval can be standardized by the unit of time used for the analysis (e.g. months) according to

$h_{jm}=-\ln(1-p_{jm})/\Delta t_{jm}$),

where ${\Delta t}_{jm}$ is the length of the interval. For the model estimation, we assigned this underlying hazard to time point $t_{m+1}$. For both outcomes the following competing survival distributions were considered using the multivariate meta-analytic framework: Weibull, Gompertz (Model 1)

| ${ln(h}_{jt})= \beta_{0j}+ \beta_{1j}t^{p}\mathrm{with}t^{0}=log(t)$ $\binom{\beta_{0j}}{\beta_{1j}}= \binom{\mu_{0jb}}{\mu_{1jb}}$  $\binom{\mu_{0jb}}{\mu_{1jb}} \sim N\left( \binom{0}{0},\left( \begin{matrix} {10}^{4} & 0 \\ 0 & {10}^{4} \end{matrix} \right) \right)$ | (1) |
| --- | --- |

and second order fractional polynomials including p_1_=0 or 1 and p_2_= -2, -1, -0.5, 0, 0.5, 1, or 2 (Model 2).

| ${ln(h}_{jt})= \left\{ \begin{aligned} \beta_{0j}+ \beta_{1j}t^{p_{1}}+\beta_{2j}t^{p_{2}} p_{1}\neq p_{2} \\ \beta_{0j}+ \beta_{1j}t^{p_{1}}+\beta_{2j}t^{p_{1}}\log\left( t \right) p_{1}= p_{2} \end{aligned} \right.\mathrm{with}t^{0}=log(t)$ $\left( \begin{matrix} \beta_{0j} \\ \beta_{1j} \\ \beta_{2j} \end{matrix} \right)= \left( \begin{matrix} \mu_{0jb} \\ \mu_{1jb} \\ \mu_{2jb} \end{matrix} \right)$  $\left( \begin{matrix} \mu_{0jb} \\ \mu_{1jb} \\ \mu_{2jb} \end{matrix} \right) \sim N\left( \left( \begin{matrix} 0 \\ 0 \\ 0 \end{matrix} \right),\left( \begin{matrix} {10}^{4} & 0 & 0 \\ 0 & {10}^{4} & 0 \\ 0 & 0 & {10}^{4} \end{matrix} \right) \right)$ | (2) |
| --- | --- |

$h_{jt}$ again reflects the underlying hazard rate in trial *j* at time point *t* and is now described as a function of time *t* with p=1 or 0 with $t^{0}=log(t)$ with treatment and study specific scale and shape parameters $\beta_{0j}$and $\beta_{1j}$. If $\beta_{1j}$ equals 0, a constant log hazard function is obtained, reflecting exponentially distributed survival times. If $\beta_{1j}$≠ 0 and *p*=1, a linear hazard function is obtained, which corresponds to a Gompertz survival function. If $\beta_{1j}$ ≠ 0 and *p*=0, a Weibull hazard function is obtained. The vectors $\binom{\mu_{0jb}}{\mu_{1jb}}$ are trial-specific and reflect the true underlying scale and shape parameters of the trial treatment. In essence, these second order fractional polynomial models are extensions of the Weibull and Gompertz model and allow arc- and bathtub shaped hazard functions, which emulate parametric distributions such as log normal and log logistic.

Table S1: Embase search strategy

| No. | Query | Results |
| --- | --- | --- |
|  | 'nonhodgkin lymphoma'/exp OR 'lymphoma'/exp | 335,237 |
|  | (('non-hodgkin*' OR nonhodgkin* OR 'non hodgkin*') NEAR/3 lymphoma*):ab,ti,kw | 58,162 |
|  | nhl:ab,ti,kw | 24,909 |
|  | #1 OR #2 OR #3 | 341,108 |
|  | indolent:ab,ti,kw OR 'low-grad*':ab,ti,kw OR 'low grad*':ab,ti,kw OR lowgrad*:ab,ti,kw OR 'slow-grow*':ab,ti,kw OR 'slow grow*':ab,ti,kw | 120,785 |
|  | #4 AND #5 | 15,066 |
|  | 'follicular lymphoma'/exp OR 'marginal zone lymphoma'/exp | 27,339 |
|  | ((follic* OR nodular*) NEAR/3 (lymph* OR blastoma*)):ab,ti,kw | 24,092 |
|  | 'brill-symmer*':ab,ti,kw OR 'brill symmer*':ab,ti,kw OR brillsymmer*:ab,ti,kw | 267 |
|  | ((centroblast* OR zentroblast* OR centrocy* OR zentrozyt* OR zentrocyt*) NEAR/1 lymph*):ab,ti,kw | 495 |
|  | 'marginal zone':ab,ti,kw OR mzl:ab,ti,kw OR 'malt lymphoma*':ab,ti,kw OR maltoma*:ab,ti,kw OR ((mucosa* NEAR/4 lymph*):ab,ti,kw) | 22,939 |
|  | #7 OR #8 OR #9 OR #10 OR #11 | 51,354 |
|  | #6 OR #12 | 59,646 |
|  | 'randomized controlled trial'/exp OR 'randomized controlled trial (topic)'/exp OR 'controlled clinical trial'/exp OR 'clinical trial'/exp OR 'multicenter study'/exp OR 'phase 2 clinical trial'/exp OR 'phase 3 clinical trial'/exp OR 'phase 4 clinical trial'/exp OR 'randomization'/exp OR 'placebo'/exp OR 'single blind procedure'/exp OR 'crossover procedure'/exp OR 'double blind procedure'/exp OR 'prospective study'/exp | 2,543,262 |
|  | 'randomi?ed controlled trial*':ab,ti,kw OR rct:ab,ti,kw OR ((random* NEAR/2 allocat*):ab,ti,kw) OR (((singl* OR doubl* OR treb* OR tripl*) NEAR/1 (blind* OR dumm* OR mask*)):ab,ti,kw) OR placebo*:ab,ti,kw | 688,550 |
|  | 'cohort analysis'/exp OR 'prospective study'/exp OR 'retrospective study'/exp OR 'longitudinal study'/exp OR 'observational study'/exp | 2,331,578 |
|  | ((prospective OR retrospective OR observational OR longitudinal OR cohort) NEAR/2 (stud* OR design*)):ti,ab,kw | 1,408,084 |
|  | #14 OR #15 OR #16 OR #17 | 4,474,421 |
|  | animal*:ab,ti,kw NOT human*:ab,ti,kw | 1,038,861 |
|  | 'animal'/exp NOT ('animal'/exp AND 'human'/exp) | 5,615,547 |
|  | 'animal model'/exp OR 'nonhuman'/exp OR 'animal experiment'/exp OR 'animal tissue'/exp | 7,037,794 |
|  | 'case report'/exp OR 'editorial'/exp OR 'historical article' | 3,365,916 |
|  | news:it OR comment:it OR editorial:it OR note:it OR 'case report':it | 1,528,861 |
|  | #19 OR #20 OR #21 OR #22 OR #23 | 13,210,579 |
|  | #18 NOT #24 | 3,932,471 |
|  | 'metastasis'/exp OR 'minimal residual disease'/exp OR 'tumor recurrence'/exp OR 'drug resistance'/exp OR 'disease exacerbation'/exp | 1,231,891 |
|  | residual*:ab,ti,kw OR malignan*:ab,ti,kw OR refractory:ab,ti,kw OR relaps*:ab,ti,kw OR recurr*:ab,ti,kw OR resist*:ab,ti,kw OR 'previously-treated':ab,ti,kw OR 'previously treated':ab,ti,kw | 3,628,697 |
|  | ((therap* OR treatment* OR chemotherap*) NEAR/3 fail*):ab,ti,kw | 137,359 |
|  | #26 OR #27 OR #28 | 4,326,394 |
|  | 'chimeric antigen receptor'/exp | 6,757 |
|  | 'chimeric antigen receptor t':ab,ti,kw OR 'anti-cd19':ab,ti,kw OR 'anti-cd 19':ab,ti,kw OR 'car t':ab,ti,kw OR cart:ab,ti,kw | 23,895 |
|  | 'axicabtagene ciloleucel'/exp | 843 |
|  | 'axicabtagene ciloleucel':ab,ti,kw OR 'kte-c19':ab,ti,kw OR yescarta*:ab,ti,kw OR 'axi-cel':ab,ti,kw | 633 |
|  | 'rituximab'/exp | 86,136 |
|  | (rituximab:ab,ti,kw OR rituxan:ab,ti,kw OR mabthera:ab,ti,kw OR 'mab-thera':ab,ti,kw OR rituzena:ab,ti,kw OR rixathon:ab,ti,kw OR idecc2b8:ab,ti,kw OR 'idec-c2b8':ab,ti,kw OR l01xc02:ab,ti,kw OR 'anti-cd20 antibody':ab,ti,kw OR anticd20:ab,ti,kw) AND antibody:ab,ti,kw | 14,603 |
|  | 'dexamethasone'/exp | 16,1427 |
|  | dexamethasone:ab,ti,kw OR dextenza:ab,ti,kw OR dexpac:ab,ti,kw | 82,579 |
|  | 'cytarabine'/exp | 64,102 |
|  | cytarabine:ab,ti,kw OR 'ara c':ab,ti,kw OR arac:ab,ti,kw | 20,534 |
|  | 'cisplatin'/exp | 192,228 |
|  | cisplatin*:ab,ti,kw OR gemcit:ab,ti,kw OR platinol*:ab,ti,kw OR platamin:ab,ti,kw OR cismaplat:ab,ti,kw OR cddp:ab,ti,kw OR biocisplatinum:ab,ti,kw OR dichlorodiammineplatinum:ab,ti,kw OR platino:ab,ti,kw OR 'platinum diamminodichloride':ab,ti,kw OR 'cis diamminedichloroplatinum':ab,ti,kw OR 'cis-diamminedichloroplatinum':ab,ti,kw OR 'cis platinum':ab,ti,kw OR 'cis-platinum':ab,ti,kw OR l01xa01:ab,ti,kw OR abiplatin:ab,ti,kw OR biocysplatinum:ab,ti,kw OR blastolem:ab,ti,kw OR briplatin:ab,ti,kw OR 'cddp ti':ab,ti,kw OR 'cis ddp':ab,ti,kw OR ((cis NEAR/3 (platinum OR platino?s OR diamine OR diaminechloroplatinum)):ab,ti,kw) OR cisplatyl:ab,ti,kw OR citoplatino:ab,ti,kw OR cytoplatin:ab,ti,kw OR cytosplat:ab,ti,kw OR docistin:ab,ti,kw OR elvecis:ab,ti,kw OR kemoplat:ab,ti,kw OR lederplatin:ab,ti,kw OR lipoplatin:ab,ti,kw OR 'mpi 5010':ab,ti,kw OR mpi5010:ab,ti,kw OR neoplatin:ab,ti,kw OR niyaplat:ab,ti,kw OR 'nk 801':ab,ti,kw OR noveldexis:ab,ti,kw OR 'nsc 119875':ab,ti,kw OR platamine:ab,ti,kw OR platiblastin:ab,ti,kw OR platidiam:ab,ti,kw OR platimine:ab,ti,kw OR platinex:ab,ti,kw OR platinil:ab,ti,kw OR platinoxan:ab,ti,kw OR ((platinum NEAR/3 (diamine OR diaminodichloride OR diamminedichloride)):ab,ti,kw) OR platiran:ab,ti,kw OR platistil:ab,ti,kw OR platistin:ab,ti,kw OR platosin:ab,ti,kw OR randa:ab,ti,kw OR romcis:ab,ti,kw OR sicatem:ab,ti,kw OR 'spi 077':ab,ti,kw OR tecnoplatin:ab,ti,kw | 104,030 |
|  | dhap:ab,ti,kw OR 'r-dhap':ab,ti,kw OR rdhap:ab,ti,kw OR dha:ab,ti,kw | 22,208 |
|  | 'ifosfamide'/exp | 31,512 |
|  | ifosfamide:ab,ti,kw OR ifomide:ab,ti,kw OR iphosphamid:ab,ti,kw OR iphosphamide:ab,ti,kw OR isoendoxan:ab,ti,kw OR 'iso-endoxan':ab,ti,kw OR isophosphamide:ab,ti,kw OR naxamide:ab,ti,kw OR ifex:ab,ti,kw OR holoxan*:ab,ti,kw OR 'ifo-cell':ab,ti,kw OR ifolem:ab,ti,kw OR ifomida:ab,ti,kw OR ifoxa:ab,ti,kw OR mitoxana:ab,ti,kw OR tronoxal:ab,ti,kw OR iff:ab,ti,kw OR ifo:ab,ti,kw OR ifx:ab,ti,kw OR ipp:ab,ti,kw OR 'mjf-9325':ab,ti,kw OR 'z-4942':ab,ti,kw | 18,903 |
|  | 'carboplatin'/exp | 73,362 |
|  | carboplatin*:ab,ti,kw OR blastocarb:ab,ti,kw OR carboplat:ab,ti,kw OR carbosin:ab,ti,kw OR carbosol:ab,ti,kw OR carbotec:ab,ti,kw OR displata:ab,ti,kw OR ercar:ab,ti,kw OR nealorin:ab,ti,kw OR novoplatinum:ab,ti,kw OR paraplatin:ab,ti,kw OR platinwas:ab,ti,kw OR ribocarbo:ab,ti,kw OR cbdca:ab,ti,kw OR 'jm-8':ab,ti,kw | 29,140 |
|  | 'etoposide'/exp | 90,794 |
|  | etoposide:ab,ti,kw OR etopophos:ab,ti,kw OR toposar:ab,ti,kw OR vepesid:ab,ti,kw OR lastet:ab,ti,kw OR epeg:ab,ti,kw OR 'vp-16':ab,ti,kw OR 'vp-16-213':ab,ti,kw | 32,525 |
|  | ice:ab,ti,kw OR ((rice NEAR/1 (chemotherap* OR regimen*)):ab,ti,kw) | 39,950 |
|  | 'bendamustine'/exp | 7,267 |
|  | bendamustin*:ab,ti,kw OR 'cep-18083':ab,ti,kw OR 'cytostasan hydrochloride':ab,ti,kw OR bendeka:ab,ti,kw OR treanda:ab,ti,kw OR levact:ab,ti,kw OR ribomustin:ab,ti,kw OR 'syb l-0501':ab,ti,kw OR 'sdx-105':ab,ti,kw | 4,319 |
|  | 'lenalidomide'/exp | 20,752 |
|  | lenalidomid*:ab,ti,kw OR 'imid 1':ab,ti,kw OR revlimid:ab,ti,kw OR 'cc 5013':ab,ti,kw OR cc5013:ab,ti,kw OR cdc501:ab,ti,kw OR 'cdc 501':ab,ti,kw | 13,103 |
|  | 'doxorubicin'/exp | 196,909 |
|  | doxorubicin:ab,ti,kw OR 'doxorubicin hydrochloride':ab,ti,kw OR aldoxorubicin:ab,ti,kw | 64,141 |
|  | 'vincristine'/exp | 104,924 |
|  | vincristine:ab,ti,kw OR 'leurocristine sulfate':ab,ti,kw OR kyocristine:ab,ti,kw | 27,260 |
|  | chop:ab,ti,kw OR rchop:ab,ti,kw | 22,265 |
|  | 'cyclophosphamide'/exp | 22,7942 |
|  | eshap:ab,ti,kw OR 'e-shap':ab,ti,kw OR reshap:ab,ti,kw | 414 |
|  | ashap:ab,ti,kw OR rashap:ab,ti,kw | 13 |
|  | gemox:ab,ti,kw OR 'gem ox':ab,ti,kw OR gemp:ab,ti,kw OR 'gem p':ab,ti,kw | 624 |
|  | 'mitoxantrone'/exp | 23,937 |
|  | mitoxantrone:ab,ti,kw OR dihydroxyanthracenedione:ab,ti,kw OR mitozantrone:ab,ti,kw OR mitroxone:ab,ti,kw OR neotalem:ab,ti,kw OR onkotrone:ab,ti,kw OR pralifan:ab,ti,kw OR dhad:ab,ti,kw OR dhaq:ab,ti,kw OR 'cl 232315':ab,ti,kw | 7,728 |
|  | 'r mice':ab,ti,kw OR rmice:ab,ti,kw OR ((mice NEAR/1 (chemotherap* OR regimen*)):ab,ti,kw) | 1,006 |
|  | 'prednisone'/exp OR 'prednisolone'/exp | 310,063 |
|  | prednisone:ab,ti,kw OR prednisolone:ab,ti,kw OR 'delta 1-cortisone':ab,ti,kw OR deltacortisone:ab,ti,kw OR deltadehydrocortisone:ab,ti,kw OR metacortandracin:ab,ti,kw OR prd:ab,ti,kw OR prednisonum:ab,ti,kw OR adasone:ab,ti,kw OR cortancyl:ab,ti,kw OR dacortin:ab,ti,kw OR decortin:ab,ti,kw OR decortisyl:ab,ti,kw OR decorton:ab,ti,kw OR deltacortene:ab,ti,kw OR deltison:ab,ti,kw OR deltra:ab,ti,kw OR econosone:ab,ti,kw OR 'meprosona f':ab,ti,kw OR ofisolona:ab,ti,kw OR panafcort:ab,ti,kw OR 'panasol s':ab,ti,kw OR paracort:ab,ti,kw OR predeltin:ab,ti,kw OR predicor:ab,ti,kw OR predicorten:ab,ti,kw OR prednicort:ab,ti,kw OR prednidib:ab,ti,kw OR prednilonga:ab,ti,kw OR predniment:ab,ti,kw OR prednitone:ab,ti,kw OR promifen:ab,ti,kw OR servisone:ab,ti,kw OR pred:ab,ti,kw | 100,779 |
|  | 'delta (1) hydrocortisone':ab,ti,kw OR 'delta1 dehydro hydrocortisone':ab,ti,kw OR deltahydrocortisone:ab,ti,kw OR metacortandralone:ab,ti,kw OR cortalone:ab,ti,kw OR hydeltra:ab,ti,kw OR hydeltrasol:ab,ti,kw OR 'meti derm':ab,ti,kw OR prelone:ab,ti,kw OR prdl:ab,ti,kw OR adnisolone:ab,ti,kw OR aprednislon:ab,ti,kw OR capsoid:ab,ti,kw OR cortisolone:ab,ti,kw OR 'dacortin h':ab,ti,kw OR decaprednil:ab,ti,kw OR 'decortin h':ab,ti,kw OR 'delta cortef':ab,ti,kw OR deltacortril:ab,ti,kw OR 'delta-diona':ab,ti,kw OR 'delta-phoricol':ab,ti,kw OR deltasolone:ab,ti,kw OR deltidrosol OR dhasolone:ab,ti,kw OR 'di adreson f':ab,ti,kw OR 'dontisolon d':ab,ti,kw OR estilsona:ab,ti,kw OR fisopred:ab,ti,kw OR frisolona:ab,ti,kw OR gupisone:ab,ti,kw OR 'hostacortin h':ab,ti,kw OR klismacort:ab,ti,kw OR kuhlprednon:ab,ti,kw OR lenisolone:ab,ti,kw OR 'lepi-cortinolo':ab,ti,kw OR 'linola-h n':ab,ti,kw OR 'linola-h-fett n':ab,ti,kw OR longiprednil:ab,ti,kw OR meticortelone:ab,ti,kw OR opredsone:ab,ti,kw OR panafcortelone:ab,ti,kw OR precortisyl:ab,ti,kw OR 'pred clysma':ab,ti,kw OR predeltilone:ab,ti,kw OR 'predni coelin':ab,ti,kw OR prednicortelone:ab,ti,kw OR 'predni helvacort':ab,ti,kw OR prenilone:ab,ti,kw | 109 |
|  | 'delta (1) hydrocortisone' OR 'delta1 dehydro hydrocortisone' OR deltahydrocortisone OR metacortandralone OR cortalone OR hydeltra OR hydeltrasol OR 'meti derm' OR prelone OR prdl OR adnisolone OR aprednislon OR capsoid OR cortisolone OR 'dacortin h' OR decaprednil OR 'decortin h' OR 'delta cortef' OR deltacortril OR 'delta diona' OR 'delta phoricol' OR deltasolone OR deltidrosol OR dhasolone OR 'di adreson f' OR 'dontisolon d' OR estilsona OR fisopred OR frisolona OR gupisone OR 'hostacortin h' OR klismacort OR kuhlprednon OR lenisolone OR 'lepi cortinolo' OR 'linola-h n' OR 'linola-h-fett n' OR longiprednil OR meticortelone OR opredsone OR panafcortelone OR precortisyl OR 'pred clysma' OR predeltilone OR 'predni coelin' OR prednicortelone OR 'predni helvacort' OR prenilone | 743 |
|  | 'idelalisib'/exp | 2,862 |
|  | idelalisib:ab,ti,kw OR 'cal 101':ab,ti,kw OR cal101:ab,ti,kw OR gs1101:ab,ti,kw OR 'gs 1101':ab,ti,kw OR zydelig:ab,ti,kw | 1,798 |
|  | 'ibrutinib'/exp | 7,691 |
|  | ibrutinib:ab,ti,kw OR 'cra 032765':ab,ti,kw OR cra032765:ab,ti,kw OR 'pci32765 00':ab,ti,kw OR pci32765:ab,ti,kw OR imbruvica:ab,ti,kw OR 'pci 32765 00':ab,ti,kw OR 'pci 32765':ab,ti,kw | 5,725 |
|  | 'copanlisib'/exp | 531 |
|  | copanlisib:ab,ti,kw OR aliqopa:ab,ti,kw OR 'bay 80 6946':ab,ti,kw OR 'bay80 6946':ab,ti,kw OR 'bay84 1236':ab,ti,kw OR 'bay 84 1236':ab,ti,kw | 304 |
|  | rgdp:ab,ti,kw OR gdp:ab,ti,kw OR rgcvp:ab,ti,kw OR gcvp:ab,ti,kw | 18,766 |
|  | 'gemcitabine'/exp | 60,545 |
|  | gemcitabine:ab,ti,kw OR gemzar:ab,ti,kw OR 'ly 188011':ab,ti,kw OR 'ly-188011':ab,ti,kw OR l01bc05:ab,ti,kw OR difluorodeoxycytidine:ab,ti,kw OR gemcite:ab,ti,kw | 30,485 |
|  | 'oxaliplatin'/exp | 42,650 |
|  | oxaliplatin:ab,ti,kw OR eloxatin:ab,ti,kw OR eloxatine:ab,ti,kw OR 'l ohp cpd':ab,ti,kw OR oxaliplatine:ab,ti,kw OR 'act 078':ab,ti,kw OR act078:ab,ti,kw | 20,632 |
|  | rcvp:ab,ti,kw OR cvp:ab,ti,kw | 6,171 |
|  | 'obinutuzumab'/exp | 2,683 |
|  | obinutuzumab:ab,ti,kw OR afutuzumab:ab,ti,kw OR gazyva:ab,ti,kw OR gazyvaro:ab,ti,kw OR ga101:ab,ti,kw OR 'ga 101':ab,ti,kw OR r7159:ab,ti,kw OR 'ro5072759 r 7159':ab,ti,kw OR 'ro 5072759':ab,ti,kw | 1,490 |
|  | 'methylprednisolone'/exp | 102,347 |
|  | methylprednisolone:ab,ti,kw OR 'adlone 40':ab,ti,kw OR 'adlone 80':ab,ti,kw OR depmedalone:ab,ti,kw OR 'depoject 80':ab,ti,kw OR depopred:ab,ti,kw OR esametone:ab,ti,kw OR firmacort:ab,ti,kw OR 'med jec 40':ab,ti,kw OR medixon:ab,ti,kw OR mednin:ab,ti,kw OR 'medralone 80':ab,ti,kw OR medrate:ab,ti,kw OR medrone:ab,ti,kw OR meprednisolone:ab,ti,kw OR meprelon:ab,ti,kw OR mesopren:ab,ti,kw OR 'methacort 40':ab,ti,kw OR methylcotol:ab,ti,kw OR methylcotolone:ab,ti,kw OR 'methylpred dp':ab,ti,kw OR methylsterolone:ab,ti,kw OR metridrol:ab,ti,kw OR metrisone:ab,ti,kw OR metycortin:ab,ti,kw OR metypred:ab,ti,kw OR metypresol:ab,ti,kw OR neomedrone:ab,ti,kw OR 'nsc 19987':ab,ti,kw OR prednol:ab,ti,kw OR solomet:ab,ti,kw OR 'solu decortin':ab,ti,kw OR medrol:ab,ti,kw OR metipred:ab,ti,kw OR urbason:ab,ti,kw | 28,298 |
|  | 'fludarabine'/exp | 29,037 |
|  | '2 fluoro 9 beta d arabinofuranosyladenine':ab,ti,kw OR '2 fluoroadenine 9 arabinoside':ab,ti,kw OR '2 fluoroadenine 9beta d arabinofuranoside':ab,ti,kw OR '2 fluoroadenine arabinofuranoside':ab,ti,kw OR 'fluoroadenine arabinoside':ab,ti,kw OR '2 fluoroara a':ab,ti,kw OR '2 fluorovidarabine':ab,ti,kw OR '9 arabinofuranosyl 2 fluoroadenine':ab,ti,kw OR '9 beta arabinofuranosyl 2 fluoroadenine':ab,ti,kw OR '9 beta d arabinofuranosyl 2 fluoroadenine':ab,ti,kw OR '9 beta dextro arabinofuranosyl 2 fluoroadenine':ab,ti,kw OR '9beta arabinofuranosyl 2 fluoroadenine':ab,ti,kw OR '9beta d arabinofuranosyl 2 fluoroadenine':ab,ti,kw OR '9beta dextro arabinofuranosyl 2 fluoroadenine':ab,ti,kw OR 'adenine,9beta dextro arabinofuranosyl 2 fluoro':ab,ti,kw OR 'arabinofuranosyl 2 fluoroadenine':ab,ti,kw OR 'arabinosyl 2 fluoroadenine':ab,ti,kw OR 'f ara a':ab,ti,kw OR 'vidarabine,2 fluoro':ab,ti,kw | 234 |
|  | 'chlorambucil'/exp | 19,123 |
|  | amboclorin:ab,ti,kw OR 'cb 1348':ab,ti,kw OR chlorambacil:ab,ti,kw OR chloraminophene:ab,ti,kw OR chlorbutin:ab,ti,kw OR chloroambucil:ab,ti,kw OR ecloril:ab,ti,kw OR leuceran:ab,ti,kw OR leukeran:ab,ti,kw OR linfolysin:ab,ti,kw OR lympholysin:ab,ti,kw OR 'nsc 3088':ab,ti,kw | 209 |
|  | supportive:ab,ti,kw AND care:ab,ti,kw OR bsc:ab,ti,kw | 53,924 |
|  | 'radiotherapy'/exp | 599,841 |
|  | ((consolidation OR adjuvant OR palliative) NEAR/1 (radiotherap* OR radiation)):ab,ti,kw | 23,026 |
|  | 'stem cell transplantation'/exp | 162,491 |
|  | autotransplant*:ab,ti,kw OR autologous:ab,ti,kw OR autograft*:ab,ti,kw OR homograft*:ab,ti,kw OR 'auto sct':ab,ti,kw OR autosct:ab,ti,kw OR asct:ab,ti,kw OR hsct:ab,ti,kw | 196,009 |
|  | (((allogenic OR allogeneic) NEAR/1 stem):ab,ti,kw) OR allosct:ab,ti,kw OR 'allo sct':ab,ti,kw | 19,882 |
|  | 'radioimmunotherapy'/exp | 5,871 |
|  | immunoradiotherapy:ab,ti,kw OR radioimmunotherapy:ab,ti,kw | 4,779 |
|  | 'watch and wait':ab,ti,kw OR 'wait and see':ab,ti,kw OR 'wait to see':ab,ti,kw | 3,888 |
|  | 'duvelisib'/exp | 585 |
|  | duvelisib:ab,ti,kw OR 'abbv 954':ab,ti,kw OR abbv954:ab,ti,kw OR copiktra:ab,ti,kw OR 'ink 1197':ab,ti,kw OR ink1197:ab,ti,kw OR 'ipi 145':ab,ti,kw OR ipi145:ab,ti,kw | 324 |
|  | 'umbralisib'/exp | 197 |
|  | umbralisib:ab,ti,kw OR 'rp 5264':ab,ti,kw OR rp5264:ab,ti,kw OR 'rp 5307':ab,ti,kw OR rp5307:ab,ti,kw OR 'tqr 1202':ab,ti,kw OR tqr1202:ab,ti,kw | 68 |
|  | 'parsaclisib'/exp | 83 |
|  | parsaclisib:ab,ti,kw OR incb050465:ab,ti,kw OR 'incb 050465':ab,ti,kw | 49 |
|  | 'abexinostat'/exp | 309 |
|  | abexinostat:ab,ti,kw OR 'cra 024781':ab,ti,kw OR cra024781:ab,ti,kw OR 'cra 024781 01':ab,ti,kw OR 'cra024781 01':ab,ti,kw OR 'pci 24781':ab,ti,kw OR pci24781:ab,ti,kw OR 'pci 24781 01':ab,ti,kw OR 'pci24781 01':ab,ti,kw OR 's 78454':ab,ti,kw OR s78454:ab,ti,kw OR 's 78454 1':ab,ti,kw | 107 |
|  | 'ibrutinib'/exp | 7,691 |
|  | ibrutinib:ab,ti,kw OR cra032765:ab,ti,kw OR 'cra 032765':ab,ti,kw OR imbruvica:ab,ti,kw OR 'pci 32765':ab,ti,kw OR pci32765:ab,ti,kw OR 'pci32765 00':ab,ti,kw OR 'pci 32765 00':ab,ti,kw | 5,725 |
|  | 'tazemetostat'/exp | 512 |
|  | tazemetostat:ab,ti,kw OR epz6438:ab,ti,kw OR 'epz 6438':ab,ti,kw | 253 |
|  | tisagenlecleucel:ab,ti,kw OR ctl019:ab,ti,kw OR 'ctl 019':ab,ti,kw OR kymriah:ab,ti,kw OR cart19:ab,ti,kw OR 'car t-19':ab,ti,kw | 883 |
|  | 'tetulomab tetraxetan lutetium lu 177'/exp | 36 |
|  | betalutin:ab,ti,kw OR '177lu dota hh1':ab,ti,kw OR '177lu dota tetulomab':ab,ti,kw OR '177lu tetraxetan tetulomab':ab,ti,kw OR 'hh1 dota lu 177':ab,ti,kw OR 'tetraxetan tetulomab lu 177':ab,ti,kw | 34 |
|  | 'odronextamab'/exp | 24 |
|  | odronextamab:ab,ti,kw OR regn1979:ab,ti,kw OR 'regn 1979':ab,ti,kw | 21 |
|  | 'me 401':ab,ti,kw | 16 |
|  | 'ibritumomab tiuxetan'/exp | 3,121 |
|  | zevalin:ab,ti,kw OR zevaline:ab,ti,kw OR ibritumomab:ab,ti,kw OR 'idec 129':ab,ti,kw OR idec129:ab,ti,kw OR 'idec y2b8':ab,ti,kw | 1,077 |
|  | 'tisagenlecleucel t'/exp | 1,196 |
|  | #30 OR #31 OR #32 OR #33 OR #34 OR #35 OR #36 OR #37 OR #38 OR #39 OR #40 OR #41 OR #42 OR #43 OR #44 OR #45 OR #46 OR #47 OR #48 OR #49 OR #50 OR #51 OR #52 OR #53 OR #54 OR #55 OR #56 OR #57 OR #58 OR #59 OR #60 OR #61 OR #62 OR #63 OR #64 OR #65 OR #66 OR #67 OR #68 OR #69 OR #70 OR #71 OR #72 OR #73 OR #74 OR #75 OR #76 OR #77 OR #78 OR #79 OR #80 OR #81 OR #82 OR #83 OR #84 OR #85 OR #86 OR #87 OR #88 OR #89 OR #90 OR #91 OR #92 OR #93 OR #94 OR #95 OR #96 OR #97 OR #98 OR #99 OR #100 OR #101 OR #102 OR #103 OR #104 OR #105 OR #106 OR #107 OR #108 OR #109 OR #110 OR #111 OR #112 OR #113 OR #114 OR #115 OR #116 OR #117 OR #118 OR #119 | 2,022,712 |
|  | #13 AND #25 AND #29 AND #120 | 4,476 |
|  | #13 AND #25 AND #29 AND #120 AND [2020-2021]/py | 398 |

Table S2: Cochrane Central Register of Controlled Trials search strategy

| No. | Query | Results |
| --- | --- | --- |
|  | MeSH descriptor: [Lymphoma] explode all trees | 3311 |
|  | MeSH descriptor: [Lymphoma, Non-Hodgkin] explode all trees | 2,044 |
|  | (('non-hodgkin*' OR nonhodgkin* OR 'non hodgkin*') NEAR/3 lymphoma):ti,ab,kw | 4,569 |
|  | ("NHL"):ti,ab,kw | 1,682 |
|  | #1 OR #2 OR #3 | 6,346 |
|  | (indolent OR 'low-grad*' OR 'low grad*' OR lowgrad* OR 'slow-grow*' OR 'slow grow*'):ti,ab,kw | 19,122 |
|  | #5 AND #6 | 819 |
|  | MeSH descriptor: [Lymphoma, Follicular] explode all trees | 330 |
|  | (((follic* OR nodular*) NEAR/3 (lymph* OR blastoma*))):ti,ab,kw | 1,472 |
|  | ('brill-symmer*' OR 'brill symmer*' OR brillsymmer*):ti,ab,kw | 0 |
|  | ((centroblast* OR zentroblast* OR centrocy* OR zentrozyt* OR zentrocyt*) NEAR/1 lymph*):ti,ab,kw | 16 |
|  | MeSH descriptor: [Lymphoma, B-Cell, Marginal Zone] explode all trees | 47 |
|  | ('marginal zone' OR mzl OR 'malt lymphoma* OR maltoma*):ti,ab,kw | 443 |
|  | (mucosa* NEAR/4 lymph*):ti,ab,kw | 248 |
|  | #8 OR #9 OR #10 OR #11 OR #12 OR #13 OR #14 | 1,938 |
|  | #7 OR #15 | 2,440 |
|  | MeSH descriptor: [Neoplasm Metastasis] explode all trees | 5,263 |
|  | MeSH descriptor: [Neoplasm, Residual] explode all trees | 327 |
|  | MeSH descriptor: [Neoplasm Recurrence, Local] explode all trees | 4,350 |
|  | MeSH descriptor: [Drug Resistance, Neoplasm] explode all trees | 556 |
|  | MeSH descriptor: [Disease Progression] explode all trees | 7,551 |
|  | (residual* OR malignan* OR refractory OR relaps* OR recurr* OR resist* OR 'previously-treated' OR 'previously treated'):ti,ab,kw | 231,263 |
|  | ((therap* OR treatment* OR chemotherap*) NEAR/3 fail*):ti,ab,kw | 35,554 |
|  | #18 OR #19 OR #20 OR #21 OR #22 OR #23 OR #24 | 264,296 |
|  | #16 AND #25 (with publication Year from 2020 to 2021, in Trials) | 89 |

Table S3: MEDLINE search strategy

| No. | Query | Hits |
| --- | --- | --- |
|  | "Lymphoma, Non-Hodgkin"[Mesh] | 104,868 |
|  | "Lymphoma"[Mesh] | 175,779 |
|  | ((nonhodgkin* or non-hodgkin*) AND lymphoma*) | 57,800 |
|  | NHL | 14,295 |
|  | #1 OR #2 OR #3 OR #4 | 186,933 |
|  | indolent or low-grad* or lowgrad* or slow-grow* | 80,162 |
|  | #5 AND #6 | 8,090 |
|  | "Lymphoma, Follicular"[Mesh] | 6,215 |
|  | ((follic* or nodular*) AND (lymph* or blastoma*)) | 35,933 |
|  | brill-symmer* or brillsymmer* | 268 |
|  | (centroblast* or zentroblast* or centrocy* or zentrozyt* or zentrocyt*) AND lymph* | 1,543 |
|  | Lymphoma, B-Cell, Marginal Zone[MeSH Terms] | 4,919 |
|  | ''marginal zone'' or MZL | 13,830 |
|  | Mucosa* AND lymph* | 41,749 |
|  | "MALT lymphoma*" or maltoma* | 3,681 |
|  | #8 OR #9 OR #10 OR #11 OR #12 OR #13 OR #14 OR #15 | 85,026 |
|  | #7 OR #16 | 89,139 |
|  | Neoplasm Metastasis[MeSH Terms] | 210,100 |
|  | Neoplasm, Residual[MeSH Terms] | 11,060 |
|  | Neoplasm Recurrence, Local[MeSH Terms] | 124,747 |
|  | Drug resistance, Neoplasm[MeSH Terms] | 53,938 |
|  | residual* or malignan* or refractory or relaps* or recurr* or resist* or "previously-treated" | 2,842,192 |
|  | (therap* or treatment* or chemotherap*) AND fail* | 702,839 |
|  | #18 OR #19 OR #20 OR #21 OR #22 OR #23 | 3,518,932 |
|  | #17 AND #24 | 28,428 |
|  | ((publisher[sb] NOT pubstatusnihms NOT pubstatuspmcsd NOT pmcbook) OR (pubstatusaheadofprint)) | 379,970 |
|  | #25 AND #26 | 171 |

Table S4: Study selection criteria to identify trials for the systematic literature review

| **PICOS** | **Criteria** |
| --- | --- |
| Population | **Inclusion:**   - Adults ≥18 years of age with R/R iNHL after failure of 2 or more lines of therapy - Patients must have received an anti-CD20 monoclonal antibody combined with an alkylating agent as prior therapy* - Patients with FL grade 1-3a - Patients with MZL nodal or extra nodal   **Exclusion:**   - Patients with R/R iNHL after first line therapy - Patients with stable iNHL (no relapse) >1 year from completion of last therapy (if unclear or not reported, then include) - Patients with FL grade 3b, spenic MZL, small lymphocytic lymphoma or lymphoplasmacytic lymphoma) or transformed FL/MZL - Patients who did not receive an anti-CD20 monoclonal antibody combined with an alkylating agent as prior therapy (if unclear or not reported, then include) |
| Intervention/ Comparator | Axicabtagene Ciloleucel (KTE-C19, axi-cel, Yescarta); Salvage chemotherapy with rituximab;  R-CHOP (rituximab, cyclophosphamide, doxorubicin, vincristine, and prednisone); R-CVP (rituximab, cyclophosphamide, vincristine, prednisolone); R-DHAP (rituximab, dexamethasone, cytarabine, cisplatin); R-DHA (rituximab, dexamethasone, and high-dose cytarabine); R-ICE (rituximab, ifosfamide, carboplatin, etoposide); R-ESHAP (rituximab, etoposide, methylprednisolone, high-dose cytarabine and cisplatin); R-GDP (gemcitabine, dexamethasone, cisplatin); R-GCVP (rituximab, gemcitabine, cyclophosphamide, vincristine, prednisolone); R-MICE (mitoxantrone, etoposide and cytarabine); Rituximab with bendamustine; Rituximab with chlorambucil; Rituximab with lenalidomide; Rituximab with fludarabine; R-GemOx (gemcitabine, oxaliplatin); R-GemP (gemcitabine, cisplatin and methylprednisolone); R-FC (fludarabine, cyclophosphamide); Rituximab monotherapy; Obinutuzumab with bendamustine or chemotherapy; Idelalisib; Ibrutinib; Copanlisib; Duvelisib; Umbralisib; Parsaclisib; Abexinostat; Imbruvica; Tazemetostat; REGN1979; Betalutin; ME-401; Kymriah; Zevalin (Ibritumomab tiuxetan); Radioimmunotherapy; Autologous or allogeneic Stem cell transplantation; Mosunetuzumab; Odronextamab; Best supportive care (including radiotherapy or wait and watch); Placebo |
| Outcomes | - Treatment response rates (cytogenetic and hematologic responses) – overall response rate (ORR), complete response (CR) and partial response (PR) - Duration of response - Progression-free survival (PFS) - Overall survival (OS) - Adverse events   - Overall   - Serious   - Severe   - Grade 3 to 4 - Withdrawals - HRQoL outcomes assessed with measures (varying measures) |
| Is Study Design | - Randomised controlled trial - Non-randomised prospective controlled clinical studies or single arm trials - Observational studies designed to measure relevant outcomes - Prospective and retrospective studies - Registries   Exclusion:   - Preclinical and phase I studies - Animal studies - Case reports - Editorials, notes, comments |
| Limits | - English and German language |

**Table S5:** Studies included in each meta-analysis

| Study | Treatment | N | 1.Main | | | | | 2. Representative cohort | | | | 3. Including off-label  (supplement only) | | | | 4. SCT (supplement only) | | | |
| --- | --- | --- | --- | --- | --- | --- | --- | --- | --- | --- | --- | --- | --- | --- | --- | --- | --- | --- | --- |
|  |  |  | Response | | | TTE curves | | Response | | TTE curves | | Response | | TTE curves | | Response | | TTE curves | |
|  |  |  | ORR | CR | OS | | PFS | OS | PFS | OS | PFS | ORR | CR | *OS** | PFS | *ORR* | *CR* | OS | PFS |
| **Batlevi 2020** | Representative cohort (MSK) | 299 |  |  | 3+ / 4+ | | 3+ / 4+ |  |  | 3+ / 4+ | 3+ / 4+ |  |  |  | 3+ |  |  |  |  |
| **Fuji 2020** | Representative cohort (Japan) | 41 | 3+ | 3+ | 3+ / 4+ | | 3+ / 4+ |  |  | 3+ / 4+ | 3+ / 4+ | 3+ | 3+ |  | 3+ |  |  |  |  |
| **Link 2019** | Representative cohort (USA) | 438 |  |  |  | | 3+ / 4+ |  |  |  | 3+ / 4+ |  |  |  | 3+ |  |  |  |  |
| **Andorsky 2019** | PI3K-d | 54 |  |  | 3+ / 4+ | | 3+ / 4+ |  |  |  |  |  |  |  | 3+ |  |  |  |  |
| **DELTA** | PI3K-d | 72 |  |  | 3+ | | 3+ |  |  |  |  |  |  |  | 3+ |  |  |  |  |
| **Robert 2019** | PI3K-d | 24 | 3+ | 3+ |  | |  |  |  |  |  | 3+ | 3+ |  |  |  |  |  |  |
| **Muntanola 2020** | Anti-CD20 combination | 28 | 3+ | 3+ |  | |  |  |  |  |  | 3+ | 3+ |  |  |  |  |  |  |
| **CHRONOS 1 Part B** | PI3K | 104 | 3+ | 3+ |  | |  |  |  |  |  | 3+ | 3+ |  |  |  |  |  |  |
| **EZH** | EZH2 | 99 | 3+ | 3+ | 3+ | | 3+ |  |  |  |  | 3+ | 3+ |  | 3+ |  |  |  |  |
| **Assouline 2020** | BsMaB | 62 | 3+ | 3+ |  | |  |  |  |  |  | 3+ | 3+ |  |  |  |  |  |  |
| **ELM-1** | BsMaB | 28 |  |  |  | |  |  |  |  |  | 3+ | 3+ |  | 3+ |  |  |  |  |
| **DAWN** | BTK | 110 |  |  |  | |  |  |  |  |  | 3+ | 3+ |  |  |  |  |  |  |
| **UNITY-NHL** | PI3K-d/CK1-e | 117 |  |  |  | |  |  |  |  |  | 3+ |  |  | 3+ |  |  |  |  |
| **Evens 2013** | SCT | 184 |  |  |  | |  |  |  |  |  |  |  |  |  |  |  | 3+ | 3+ |
| **Ito 2013** | SCT | 30 |  |  |  | |  |  |  |  |  |  |  |  |  |  |  | 3+ | 3+ |
| **Khouri 2008** | SCT | 47 |  |  |  | |  |  |  |  |  |  |  |  |  |  |  | 3+ | 3+ |
| **Laport 2016** | SCT | 62 |  |  |  | |  |  |  |  |  |  |  |  |  |  |  | 3+ | 3+ |
| **Lunning 2016** | SCT | 44 |  |  |  | |  |  |  |  |  |  |  |  |  |  |  | 3+ |  |
| **Sesques 2020** | SCT | 61 |  |  |  | |  |  |  |  |  |  |  |  |  |  |  | 3+ | 3+ |
| **Vose 2008** | SCT | 108 |  |  |  | |  |  |  |  |  |  |  |  |  |  |  | 3+ |  |

| 3+ | Contributes to 3^rd^ line plus model | 3+ / 4+ | Contributes to 3^rd^ line plus and 4^th^ line plus models |  | Data is available but model is redundant or has too few data |  | Study not eligible for inclusion in model |
| --- | --- | --- | --- | --- | --- | --- | --- |

* Model includes same studies as main model, so is redundant. MSK, Memorial Sloan Kettering cancer center; R-ESHAP, rituximab plus etoposide, cytarabine, cisplatinum and methylprednisolone; SCT, stem cell transplant; PI3K, phosphatidylinositol 3-kinase; BsMAb Bispecific monoclonal antibody; PI3K-d/CK1-e phosphatidylinositol 3-kinase-delta and casein kinase-1 epsilon; EZH2 Enhancer of zeste homolog 2

**Table S6: Study quality assessment results**

|  |  |  | **Batlevi, 2020** | **Link, 2019** | **Fuji, 2020** | **Andorsky 2019** | **Assouline 2020** | **DAWN** | **Robert 2019** | **Evens 2013** | **Lunning 2016** | **Vose 2008** | **Ito 2013** | **Khouri 2008** | **Laport 2016** | **ELM-1** | **UNITY-NHL** | **EZH** | **Wagner-Johnston 2021** | **CHRONOS 1 Part B** | **DELTA** | **Muntanola 2020** |
| --- | --- | --- | --- | --- | --- | --- | --- | --- | --- | --- | --- | --- | --- | --- | --- | --- | --- | --- | --- | --- | --- | --- |
| 1 | Is the hypothesis/aim/objective of the study clearly described? | Yes=1; No=0 | 1 | 1 | 1 | 1 | 1 | 1 | 1 | 1 | 1 | 1 | 1 | 1 | 1 | 1 | 1 | 1 | 1 | 1 | 1 | 1 |
| 2 | Are the main outcomes to be measured clearly described in the introduction or methods section? If the main outcomes are first mentioned in the Results section, the question should be answered no. All primary outcomes should be described for YES | Yes=1; No=0 | 1 | 1 | 1 | 1 | 1 |  | 1 | 1 | 1 | 1 | 1 | 1 | 1 | 1 | 1 | 1 | 1 | 1 | 1 | 0 |
| 3 | Are the characteristics of the patients included in the study clearly described? In cohort studies and trials, inclusion and/or exclusion criteria should be given. In case-control studies, a case-definition and the source for controls should be given. Single case studies must state source of patient | Yes=1; No=0 | 1 | 1 | 1 | 1 | 1 | 1 | 0 | 1 | 1 | 1 | 1 | 1 | 1 | 1 | 1 | 1 | 1 | 1 | 1 | 1 |
| 4 | Are the interventions of interest clearly described? Treatments and placebo (where relevant) that are to be compared should be clearly described. Treatments and placebo (where relevant) that are to be compared should be clearly described. | Yes=1; No=0 | 1 | 1 | 1 | 1 | 1 | 1 | 1 | 1 | 1 | 1 | 1 | 1 | 1 | 1 | 1 | 1 | 1 | 1 | 1 | 1 |
| 5 | Are the distributions of principal confounders in each group of patients to be compared clearly described? A list of principal confounders is provided | Yes=1; No=0 | 0 | 0 | 0 | 1 | 0 | 1 | 0 | 1 | 0 | 0 | 1 | 0 | 1 | 1 | 1 | 1 | 1 | 1 | 1 | 0 |
| 6 | Are the main findings of the study clearly described? Simple outcome data (including denominators and numerators) should be reported for all major findings so that the reader can check the major analyses and conclusions. (This question does not cover statistical tests that are considered below). | Yes=1; No=0 | 1 | 1 | 1 | 1 | 1 | 1 | 1 | 1 | 1 | 1 | 1 | 1 | 1 | 1 | 1 | 1 | 1 | 1 | 1 | 1 |
| 7 | Does the study provide estimates of the random variability in the data for the main outcomes? In non-normally distributed data, the inter-quartile range of results should be reported. In normally distributed data, the standard error, standard deviation, or confidence intervals should be reported. If the distribution of the data is not described, it must be assumed that the estimates used were appropriate and the question should be answered yes | Yes=1; No=0 | 1 | 1 | 1 | 1 | 1 | 1 | 0 | 1 | 1 | 1 | 1 | 1 | 1 | 1 | 1 | 1 | 1 | 1 | 1 | 1 |
| 8 | Have all important adverse events that may be a consequence of the intervention been reported? This should be answered yes if the study demonstrates that there was a comprehensive attempt to measure adverse events. | Yes=1; No=0 | 0 | 0 | 0 | 1 | 1 | 1 | 0 | 0 | 0 | 0 | 1 | 1 | 1 | 1 | 1 | 1 | 1 | 1 | 1 | 0 |
| 9 | Have the characteristics of patients lost to follow-up been described? This should be answered yes where there were no losses to follow-up or where losses to follow-up were so small that findings would be unaffected by their inclusion. This should be answered no when a study does not report the number of patients lost to follow-up | Yes=1; No=0 | 1 | 0 | 0 | 0 | 1 | 1 | 1 | 1 | 0 | 0 | 1 | 0 | 0 | 0 | 1 | 0 | 1 | 1 | 0 | 0 |
| 10 | Have actual probability values been reported (e.g., 0.035 rather than <0.001? If no p values are presented, the question should be answered ‘no’. If values presented and there is a mixture of reporting (some presented as < or > specific figures, some as equality, e.g.=0.034), question should be answered ‘yes’. | Yes=1; No=0 | 1 | 0 | 1 | 0 | 1 | 1 | 0 | 1 | 0 | 1 | 1 | 1 | 1 | 0 | 0 | 0 | 0 | 0 | 0 | 0 |
| 11 | Were the subjects asked to participate in the study representative of the entire population from which they were recruited? The study must identify the source population for patients and describe how the patients were selected. Patients would be representative if they comprised the entire source population, an unselected sample of consecutive patients, or a random sample. Random sampling is only feasible where a list of all members of the relevant population exists. Where a study does not report the proportion of the source population from which the patients are derived, the question should be answered as unable to determine. | Yes=1; No=0; Unable to determine=0 | 1 | 1 | 1 | 1 | 1 | 1 | 1 | 1 | 1 | 1 | 1 | 1 | 1 | 1 | 1 | 1 | 1 | 1 | 1 | 0 |
| 12 | Were those subjects who were prepared to participate representative of the entire population from which they were recruited? The proportion of those asked and agreed to participate should be stated. Validation that the sample was representative would include demonstrating that the distribution of the main confounding factors was the same in the study sample and the source population | Yes=1; No=0; Unable to determine=0 | 1 | 0 | 1 | 0 | 0 | 1 | 0 | 1 | 0 | 0 | 1 | 0 | 1 | 0 | 0 | 0 | 0 | 1 | 1 | 0 |
| 13 | Were the staff, places, and facilities where the patients were treated representative of the treatment the majority of patients receive? For the question to be answered yes the study should demonstrate that the intervention was representative of that in use in the source population. The question should be answered no if, for example, the intervention was undertaken in a specialist center unrepresentative of the hospitals most of the source population would attend. | Yes=1; No=0; Unable to determine=0 | 1 | 1 | 1 | 1 | 1 | 1 | 0 | 0 | 1 | 0 | 0 | 1 | 1 | 0 | 0 | 0 | 0 | 1 | 1 | 0 |
| 14 | 14. Was an attempt made to blind study subjects to the intervention they have received? For studies where the patients would have no way of knowing which intervention they received, this should be answered yes | Yes=1; No=0; Unable to determine=0 | 0 | 0 | 0 | 0 | 0 | 0 | 0 | 0 | 0 | 0 | 0 | 0 | 0 | 0 | 0 | 0 | 0 | 0 | 0 | 0 |
| 15 | Was an attempt made to blind those measuring the main outcomes of the intervention? | Yes=1; No=0; Unable to determine=0 | 0 | 0 | 0 | 0 | 0 | 0 | 0 | 0 | 0 | 0 | 0 | 0 | 0 | 0 | 0 | 0 | 0 | 0 | 0 | 0 |
| 16 | If any of the results of the study were based on “data dredging,” was this made clear? Any analyses that had not been planned at the outset of the study should be clearly indicated. If no retrospective unplanned subgroup analyses were reported, then answer yes. | Yes=1; No=0; Unable to determine=0 | 0 | 1 | 1 | 1 | 1 | 1 | 0 | 0 | 1 | 1 | 0 | 1 | 0 | 0 | 0 | 0 | 0 | 1 | 1 | 0 |
| 17 | In trials and cohort studies, do the analyses adjust for different lengths of follow-up of patients, or in case-control studies, is the time period between the intervention and outcome the same for cases and controls? Where follow-up was the same for all study patients, the answer should yes. If different lengths of follow-up were adjusted, for example, by survival analysis, the answer should be yes. Studies where differences in follow-up are ignored should be answered no | Yes=1; No=0; Unable to determine=0 | 1 | 1 | 1 | 1 | 1 | 1 | 1 | 1 | 1 | 1 | 1 | 1 | 1 | 0 | 0 | 0 | 1 | 1 | 1 | 0 |
| 18 | Were the statistical tests used to assess the main outcomes appropriate? The statistical techniques used must be appropriate to the data. For example nonparametric methods should be used for small sample sizes. Where little statistical analysis has been undertaken but where there is no evidence of bias, the question should be answered yes. If the distribution of the data (normal or not) is not described it must be assumed that the estimates used were appropriate and the question should be answered yes. | Yes=1; No=0; Unable to determine=0 | 1 | 1 | 1 | 1 | 1 | 1 | 0 | 1 | 1 | 1 | 1 | 1 | 1 | 1 | 1 | 1 | 1 | 1 | 1 | 1 |
| 19 | Was compliance with the intervention(s) reliable? Where there was noncompliance with the allocated treatment or where there was contamination of one group, the question should be answered no. For studies where the effect of any misclassification was likely to bias any association to the null, the question should be answered yes. | Yes=1; No=0; Unable to determine=0 | 0 | 0 | 0 | 1 | 1 | 1 | 1 | 1 | 1 | 1 | 1 | 1 | 1 | 0 | 0 | 0 | 0 | 1 | 1 | 0 |
| 20 | Were the main outcome measures used accurate (valid and reliable)? For studies where the outcome measures are clearly described, the question should be answered yes. For studies that refer to other work or demonstrates the outcome measures are accurate, the question should be answered as yes. | Yes=1; No=0; Unable to determine=0 | 1 | 1 | 1 | 1 | 1 | 1 | 1 | 1 | 1 | 1 | 1 | 1 | 1 | 1 | 1 | 1 | 1 | 1 | 1 | 1 |
| 21 | Were the patients in different intervention groups (trials and cohort studies) or were the cases and controls (case-control studies) recruited from the same population? For example, patients for all comparison groups should be selected from the same hospital. The question should be answered “unable to determine” for cohort and case control studies where there is no information concerning the source of patients included in the study | Yes=1; No=0; Unable to determine=0 | 1 | 1 | 1 | 0 | 0 | 0 | 0 | 0 | 0 | 1 | 1 | 0 | 0 | 0 | 0 | 0 | 0 | 0 | 0 | 0 |
| 22 | Were study subjects in different intervention groups (trials and cohort studies) or were the cases and controls (case-control studies) recruited over the same period of time? For a study which does not specify the time period over which patients were recruited, the question should be answered as “unable to determine.” | Yes=1; No=0; Unable to determine=0 | 1 | 1 | 1 | 1 | 0 | 1 | 0 | 0 | 1 | 1 | 0 | 1 | 1 | 0 | 1 | 0 | 0 | 1 | 0 | 0 |
| 23 | Were study subjects randomised to intervention groups? Studies that state that subjects were randomised should be answered yes except where method of randomisation would not ensure random allocation. For example, alternate allocation would score 0 because it is predictable | Yes=1; No=0; Unable to determine=0 | 0 | 0 | 0 | 0 | 0 | 0 | 0 | 0 | 0 | 0 | 0 | 0 | 0 | 0 | 0 | 0 | 0 | 0 | 0 | 0 |
| 24 | Was the randomised intervention assignment concealed from both patients and healthcare staff until recruitment was complete and irrevocable? All nonrandomised studies should be answered no. If assignment was concealed from patients but not from staff, it should be answered no. | Yes=1; No=0; Unable to determine=0 | 0 | 0 | 0 | 0 | 0 | 0 | 0 | 0 | 0 | 0 | 0 | 0 | 0 | 0 | 0 | 0 | 0 | 0 | 0 | 0 |
| 25 | Was there adequate adjustment for confounding in the analyses from which the main findings were drawn? This question should be answered no for trials if: the main conclusions of the study were based on analyses of treatment rather than intention-to-treat; the distribution of known confounders in the different treatment groups was not described; or the distribution of known confounders differed between the treatment groups but was not taken into account in the analyses. In nonrandomised studies, if the effect of the main confounders was not investigated or confounding was demonstrated but no adjustment was made in the final analyses, the question should be answered as no. | Yes=1; No=0; Unable to determine=0 | 0 | 0 | 0 | 0 | 0 | 0 | 0 | 0 | 0 | 1 | 0 | 0 | 0 | 0 | 0 | 0 | 0 | 0 | 0 | 0 |
| 26 | Were losses of patients to follow-up taken into account? If the numbers of patients lost to follow-up are not reported, the question should be answered as “unable to determine.” If the proportion lost to follow-up was too small to affect the main findings, the question should be answered yes | Yes=1; No=0; Unable to determine=0 | 1 | 1 | 0 | 0 | 0 | 1 | 0 | 1 | 0 | 0 | 1 | 0 | 0 | 0 | 1 | 0 | 0 | 1 | 0 | 0 |
|  | **Total** |  | **17** | **15** | **16** | **16** | **16** | **19** | **9** | **16** | **14** | **16** | **18** | **16** | **17** | **11** | **14** | **11** | **13** | **19** | **16** | **7** |

**Table S7: Response criteria used for each study included in the meta-analysis**

| **Study** | **Year** | **Treatment** | **Response criteria if reported** |
| --- | --- | --- | --- |
| Andorsky 2019 | 2019 | Idelalisib | -- |
| Assouline 2020 | 2020 | Mosunetuzumab | Standard Criteria for NHL |
| Batlevi 2020 | 2020 | Representative cohort | -- |
| CHRONOS 1 part B | 2017 | Copanlisib | 2007 IWG-NHL criteria |
| DAWN | 2018 | Ibrutinib | 2007 IWG-NHL criteria |
| DELTA | 2014 | Idelalisib | 2007 IWG-NHL criteria |
| ELM-1 | 2020 | Odronextamab | -- |
| Evens 2013 | 2013 | Allo-SCT | -- |
| EZH | 2018 | Tazemetostat | 2007 IWG-NHL criteria |
| Fuji 2020 | 2020 | Representative cohort | -- |
| Ito 2013 | 2013 | Allo-SCT | -- |
| Khouri 2008 | 2008 | Allo-SCT | 1999 IWG-NHL criteria |
| Laport 2016 | 2016 | Allo-SCT, Auto SCT | 2007 IWG-NHL criteria |
| Link 2019 | 2019 | Representative cohort | -- |
| Lunning 2016 | 2016 | Auto-SCT | 2007 IWG-NHL criteria |
| Muntanola 2020 | 2020 | R-ESHAP | 1999 IWG-NHL criteria |
| Robert 2019 | 2019 | Idelalisib | -- |
| Sesques 2020 | 2020 | Auto-SCT | 1999 IWG-NHL criteria or 2007 IWG-NHL criteria |
| UNITY-NHL | 2021 | Umbralisib | Lugano classification |
| Vose 2008 | 2008 | Auto-SCT | 1999 IWG-NHL criteria |

**Table S8: Meta-analysis of response outcomes, separated by treatment category**

**a. Main analysis**

| **Treatment** | **N patients** | **N studies** | **Fixed-effect Estimates**  **% (95% CI)** | **Random-effects Estimates**  **% (95% CI)** | **I^2^** |
| --- | --- | --- | --- | --- | --- |
| **Overall response rate** | | | | | |
| Anti-CD20 combination | 28 | 1 | 71.43  (53.62 - 86.33) | 71.43  (53.62 - 86.33) | -- |
| BsMAb | 62 | 1 | 67.74  (55.68 - 78.71) | 67.74  (55.68 – 78.71) |  |
| EZH2 inhibitor | 99 | 1 | 51.52  (41.7 – 61.27) | 51.52  (41.7 – 61.27) | -- |
| PI3k-d inhibitor | 200 | 3 | 60.82  (53.97 – 67.46) | 64.22  (50.06 – 77.21) | 44.1  (0.6 – 63.18) |
| Representative cohort | 85 | 4 | 45.29  (34.89 – 55.9) | 41.28  (25.04 – 58.56) | 28.94  (0 – 57.11) |
| **Overall** | **412** | **10** | **57.72  (53.25 - 62.13)** | **58.47  (51.13 – 65.62)** |  |
| **Complete response** | | | | | |
| Anti-CD20 combination | 28 | 1 | 46.43  (28.62 – 64.72) | 46.43  (28.62 – 64.72) | -- |
| BsMAb | 62 | 1 | 50  (37.68 – 62.32) | 50  (37.68 – 62.32) |  |
| EZH2 inhibitor | 99 | 1 | 6.06  (2.24 – 11.58) | 6.06  (2.24 – 11.58) | -- |
| PI3k-d inhibitor | 200 | 3 | 17.92  (12.93 – 23.53) | 17.92  (12.93 – 23.53) | 0  (0 – 60.97) |
| Representative cohort | 85 | 4 | 24.28  (15.81 – 33.91) | 16.72  (2.83 – 38.91) | 50.97  (19.31 – 63.59) |
| **Overall** | **412** | **9** | **20.98  (17.44 - 24.76)** | **19.63  (15.02 - 24.68)** |  |

Treatment-class estimates should be interpreted with caution due to the small sample size within each category. BsMAb, bispecific monoclonal antibody; CI, confidence interval; EZH2. Enhancer of zeste homolog 2; PI3k-d Phosphoinositide 3-kinase delta.

**b. With inclusion of off-label treatments**

| **Treatment** | **N patients** | **N studies** | **Fixed-effect Estimates**  **% (95% CI)** | **Random-effects Estimates**  **% (95% CI)** | **I^2^** |
| --- | --- | --- | --- | --- | --- |
| **Overall response rate** | | | | | |
| Anti-CD20 combination | 28 | 1 | 71.43  (53.62 - 86.33) | 71.43  (53.62 - 86.33) | -- |
| BsMAb | 90 | 2 | 76.99  (67.78 - 85.05) | 81.5  (51.43 - 98.61) | -- |
| BTK inhibitor | 110 | 1 | 20.91  (13.86 - 28.97) | 20.91  (13.86 - 28.97) | -- |
| EZH2 inhibitor | 99 | 1 | 51.52  (41.7 - 61.27) | 51.52  (41.7 - 61.27) | -- |
| PI3k-d / CK1-e inhibitor | 185 | 2 | 46.48  (39.36 - 53.68) | 46.48  (39.36 - 53.68) | -- |
| PI3k-d inhibitor | 200 | 3 | 60.82  (53.97 - 67.46) | 64.22  (50.06 - 77.21) | 44.1  (0.6 - 63.18) |
| Representative cohort | 85 | 4 | 45.29  (34.89 - 55.9) | 41.28  (25.04 - 58.56) | 28.94  (0 - 57.11) |
| **Overall** | **797** | **14** | **51.32**  **(47.85 - 54.78)** | **52.40**  **(46.37 - 58.39)** |  |
| **Complete response** | | | | | |
| Anti-CD20 combination | 28 | 1 | 46.43  (28.62 - 64.72) | 46.43  (28.62 - 64.72) | -- |
| BsMAb | 90 | 2 | 58.11  (47.82 - 68.06) | 62.04  (36.73 - 84.26) | -- |
| BTK inhibitor | 110 | 1 | 10.91  (5.8 - 17.38) | 10.91  (5.8 - 17.38) | -- |
| EZH2 inhibitor | 99 | 1 | 6.06  (2.24 - 11.58) | 6.06  (2.24 - 11.58) | -- |
| PI3k-d / CK1-e inhibitor | 117 | 1 | 5.13  (1.89 - 9.84) | 5.13  (1.89 - 9.84) | -- |
| PI3k-d inhibitor | 200 | 3 | 17.92  (12.93 – 23.53) | 17.92  (12.93 – 23.53) | 0  (0 – 60.97) |
| Representative cohort | 85 | 4 | 24.28  (15.81 - 33.91) | 16.72  (2.83 - 38.91) | 50.97  (19.31 - 63.59) |
| **Overall** | **729** | **13** | **17.91**  **(15.22 - 20.78)** | **17.46**  **(13.59 - 21.70)** |  |

Treatment-class estimates should be interpreted with caution due to the small sample size within each category. BsMAb, bispecific monoclonal antibody; CI, confidence interval; EZH2. Enhancer of zeste homolog 2; PI3k-d Phosphoinositide 3-kinase delta.

**Table S9: Model selection across the Bayesian analyses**

| **Population** | **Overall Survival** | | **Progression-free Survival** | |
| --- | --- | --- | --- | --- |
|  | **Model** | **Parameters** | **Model** | **Parameters** |
| Main ≥3^rd^ line | Fixed-effect | P_1_ = 0; P_2_ = 0 | Fixed-effect | P_1_ = 0; P_2_ = 0 |
| Main ≥4^th^ line | Fixed-effect | P_1_ = 0; P_2_ = 0 | Fixed-effect | P_1_ = 0; P_2_ = -1 |
| Representative cohorts ≥3^rd^ line | Fixed-effect | P_1_ = 0; P_2_ = -0.5 | Fixed-effect | P_1_ = 0; P_2_ = 0 |
| Representative cohorts ≥4^th^ line | Fixed-effect | P_1_ = 0; P_2_ = 0 | Fixed-effect | P_1_ = 0; P_2_ = -1 |
| Including off-label treatments, ≥3^rd^ line |  |  | Fixed-effect | P_1_ = 0; P_2_ = 0 |
| SCT only, ≥3^rd^ line | Fixed-effect | P_1_ = 1; P_2_ = 0 | Fixed-effect | P_1_ = 0; P_2_ = -2 |

**Table S10: Time-to-event meta-analysis results for supplemental models**

|  | **Pooled Kaplan-Meier analyses** | | **Bayesian fractional polynomial meta-analyses** | |
| --- | --- | --- | --- | --- |
|  | **Median OS**  **(95% CI)** | **Median PFS**  **(95% CI)** | **Median OS**  **(95% CrI)** | **Median PFS**  **(95% CrI)** |
| Including off-label, ≥3^rd^ line | NA | 10.20  (9.48, 11.10) | NA | 9.86  (9.16 - 10.67) |
| SCT only ≥3^rd^ line | 90.35  (78.32, 120.96) | 41.26  (32.19, 52.67) | 93.9  (81.8 - 107.96) | 38.58  (31.37 - 47.94) |
|  | **OS at 18m**  **(95% CI)** | **PFS at 18m**  **(95% CI)** | **OS at 24m**  **(95% CI)** | **PFS at 24m**  **(95% CI)** |
| Including off-label, ≥3^rd^ line | NA | 35.18  (33.02, 37.47) | NA | 27.94  (25.89, 30.16) |
| SCT only ≥3^rd^ line | 83.52  (80.69, 86.46) | 66.10  (61.79, 70.71) | 78.75  (75.59, 82.06) | 59.92  (55.45, 64.76) |

**Figure S1: Meta-analysis of response outcomes, including off-label treatments**

**A) Overall response rate**

**
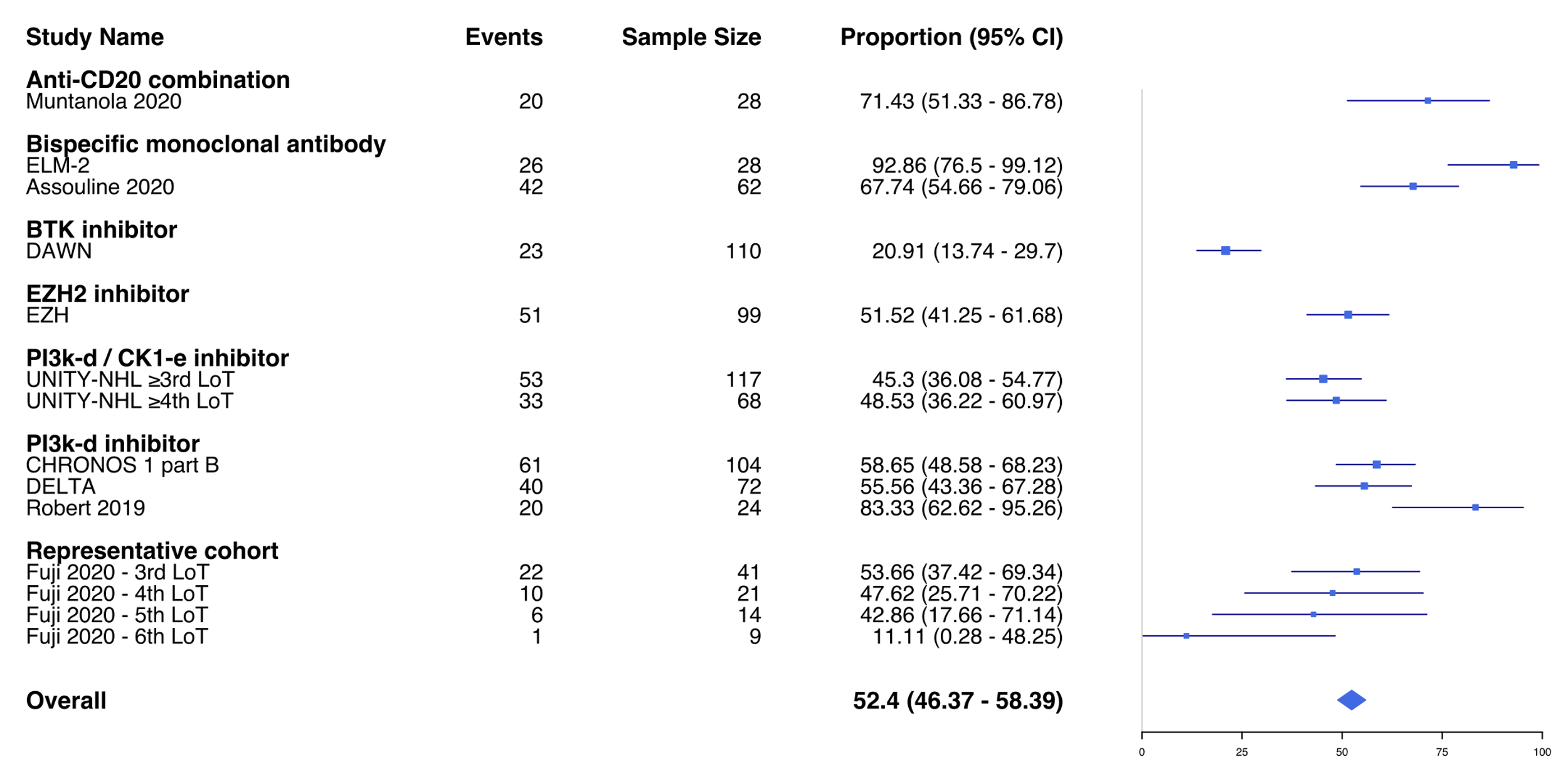
**

**B) Complete response**


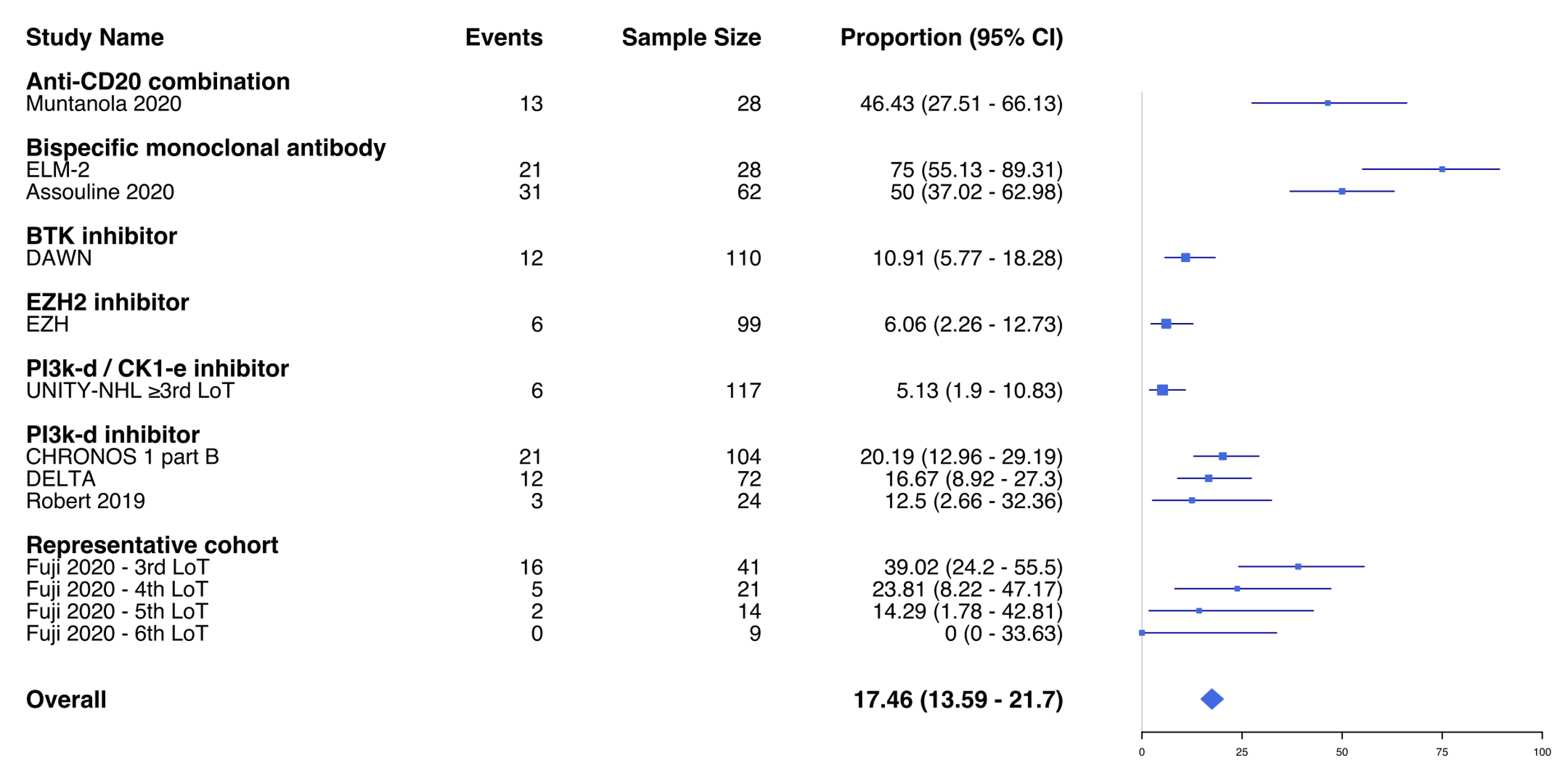


**Figure S2: Pooled KM curves for supplemental model including off-label treatments**

A) PFS 3^rd^ line plus B) OS 3^rd^ line plus


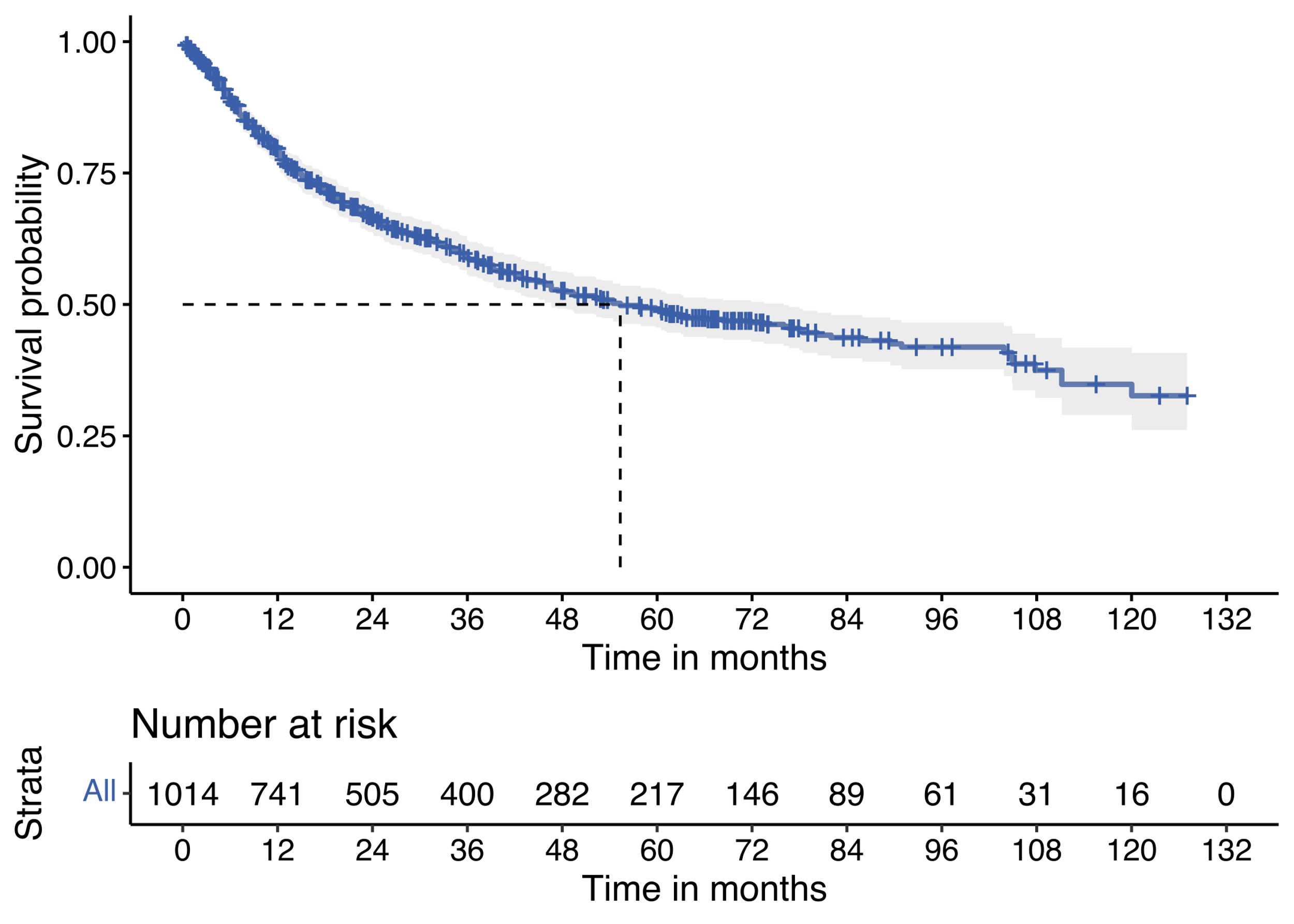


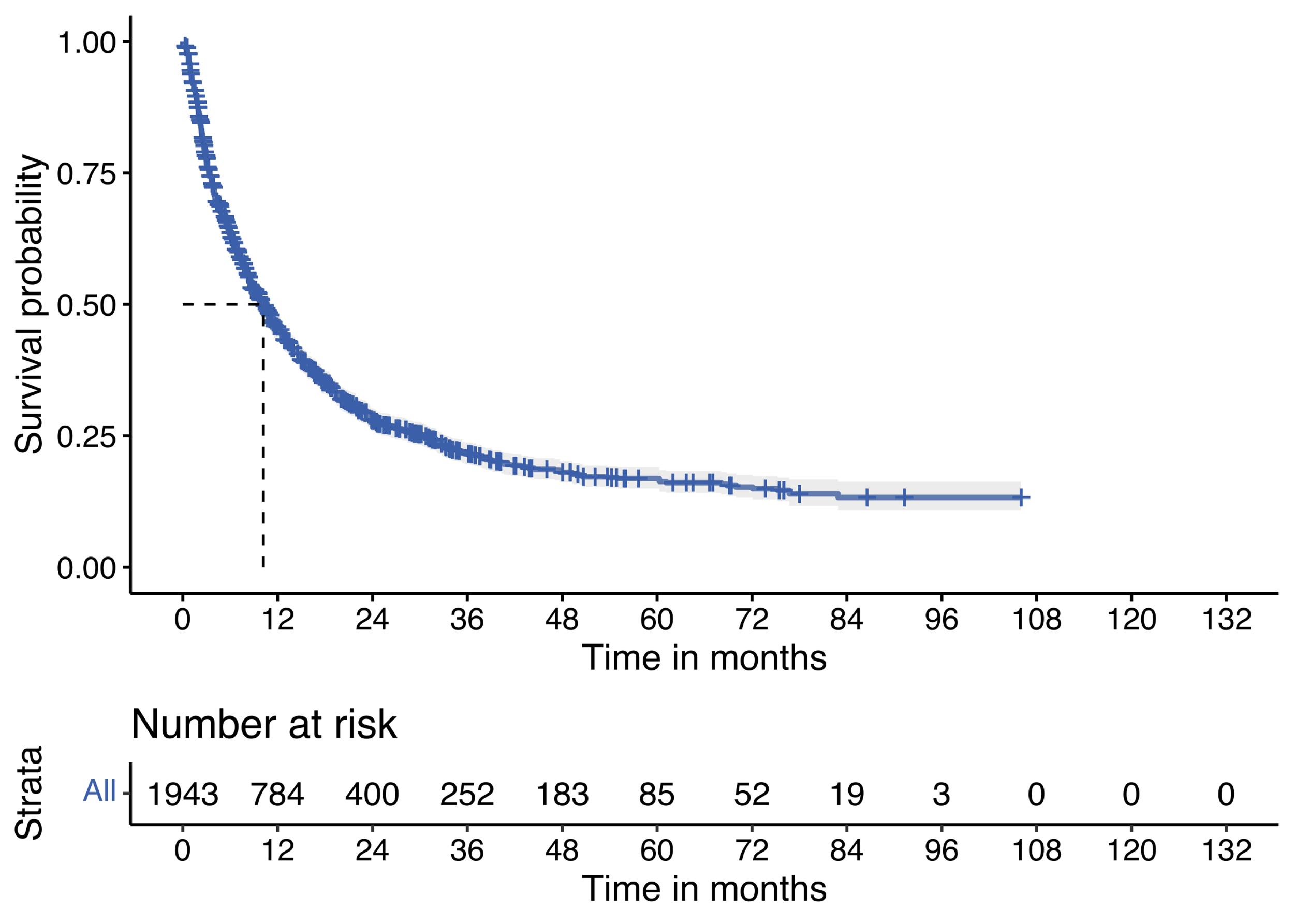


**Figure S3: Pooled KM curves for supplemental model including only SCT studies**

A) PFS 3^rd^ line plus B) OS 3^rd^ line plus


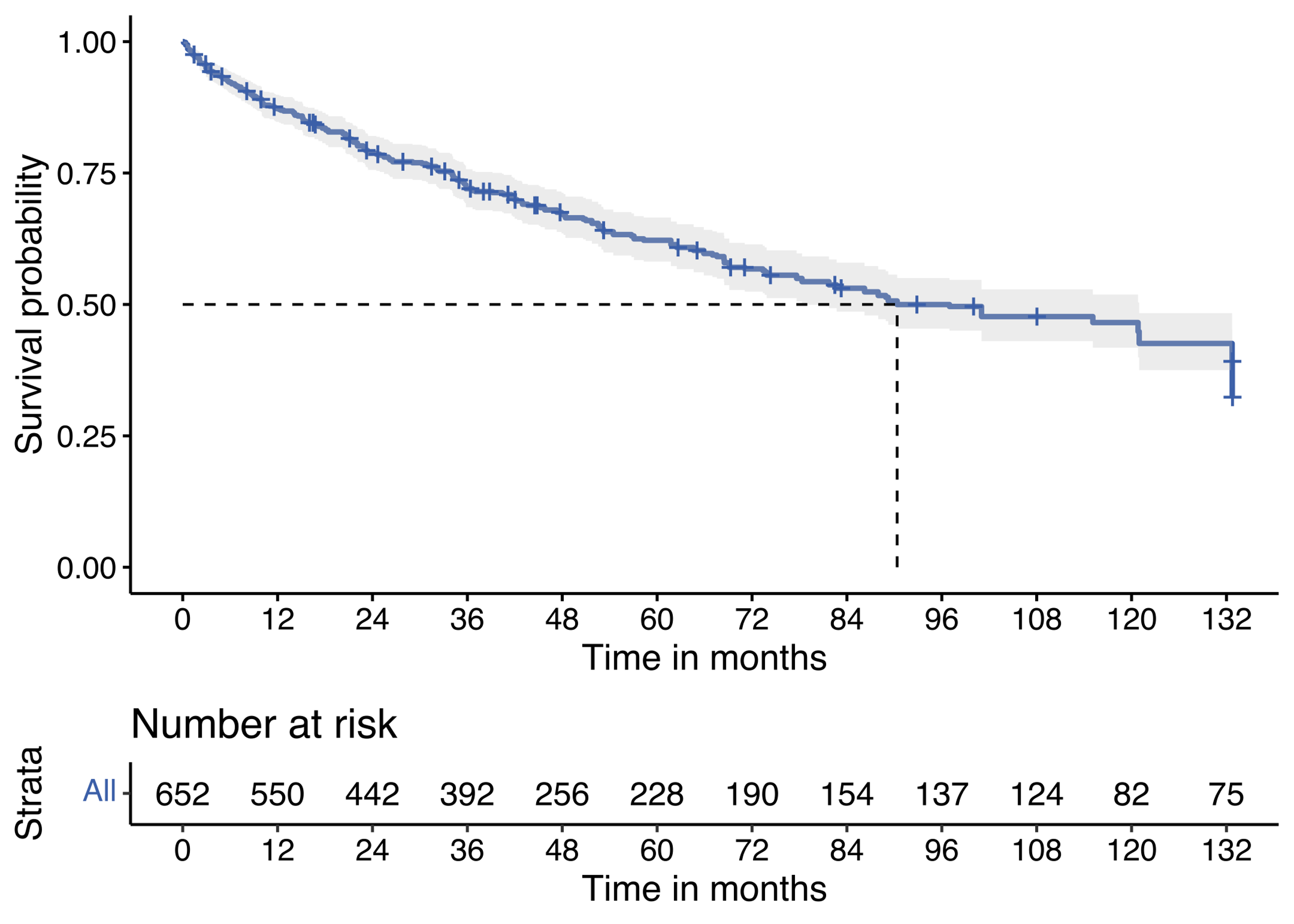


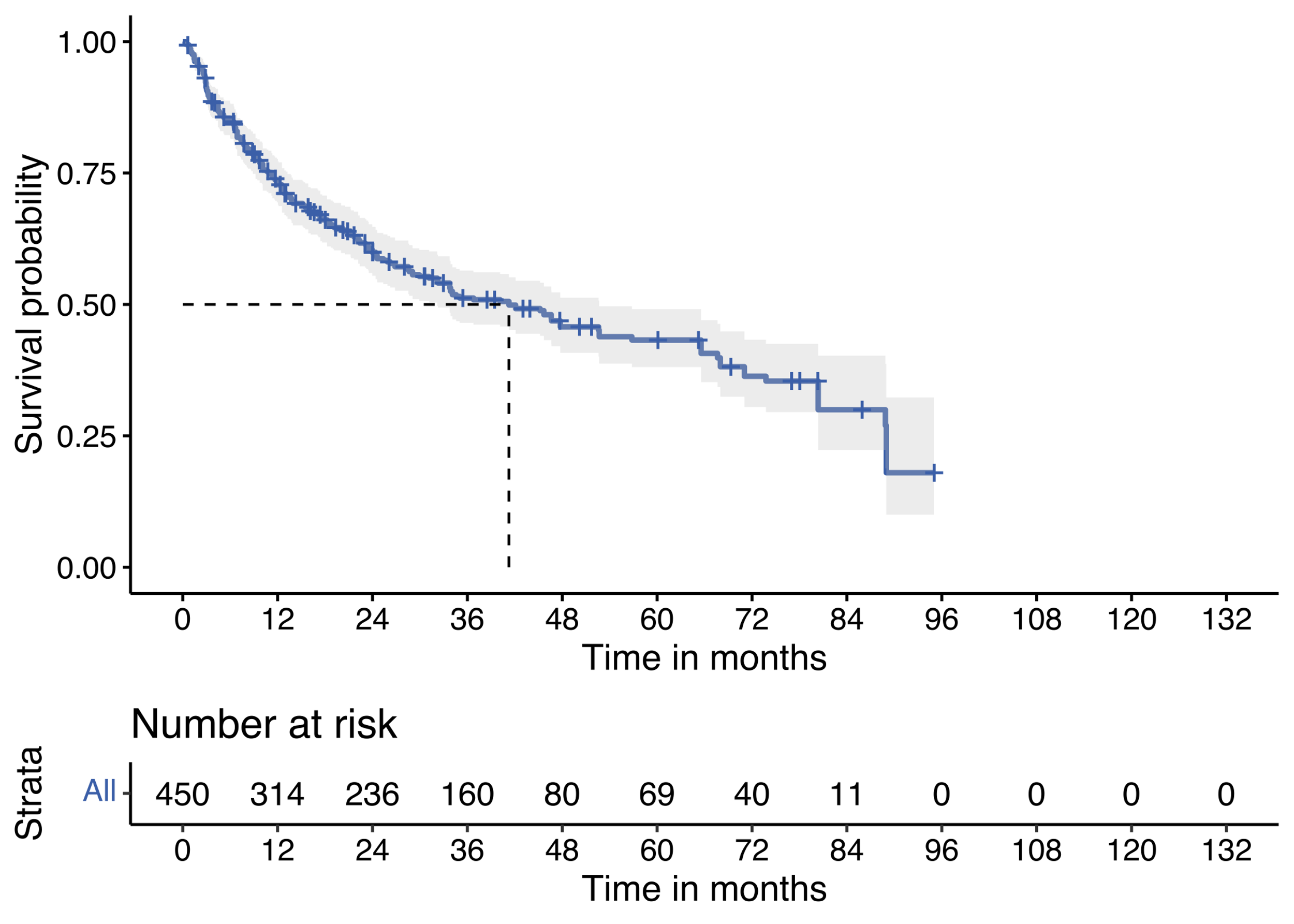

Supplement: Supplementary file 1 — Additional file 1: Table S1. Embase search strategy. Table S2. Cochrane Central Register of Controlled Trials search strategy. Table S3. MEDLINE search strategy. Table S4. Study selection criteria to identify trials for the systematic literature review. Table S5. Studies included in each meta-analysis. Table S6. Study quality assessment results. Table S7. Response criteria used for each study included in the meta-analysis. Table S8. Meta-analysis of response outcomes, separated by treatment category, a. Main analysis, b. With inclusion of off-label treatments. Table S9. Model selection across the Bayesian analyses. Table S10. Time-to-event meta-analysis results for supplemental models. Fig. S1. Meta-analysis of response outcomes, including off-label treatments, A) Overall response rate, B) Complete response. Fig. S2. Pooled KM curves for supplemental model including off-label treatments, A) PFS 3rd line plus, B) OS 3rd line plus, Fig. S3. Pooled KM curves for supplemental model including only SCT studies, A) PFS 3rd line plus, B) OS 3rd line plus. [file 12885_2023_10546_MOESM1_ESM.docx]
